# TRPM7 kinase is required for insulin production and compensatory islet responses during obesity

**DOI:** 10.1172/jci.insight.163397

**Published:** 2023-02-08

**Authors:** Noushafarin Khajavi, Andreas Beck, Klea Riçku, Philipp Beyerle, Katharina Jacob, Sabrina F. Syamsul, Anouar Belkacemi, Peter S. Reinach, Pascale C.F. Schreier, Houssein Salah, Tanja Popp, Aaron Novikoff, Andreas Breit, Vladimir Chubanov, Timo D. Müller, Susanna Zierler, Thomas Gudermann

**Affiliations:** 1 Walther Straub Institute of Pharmacology and Toxicology, LMU Munich, Munich, Germany.; 2 Institute of Experimental and Clinical Pharmacology and Toxicology, Saarland University, Homburg, Germany.; 3 Wenzhou Medical University, Ophthalmology Department, Wenzhou, China.; 4 Bundeswehr Institute of Radiobiology, Munich, Germany.; 5 Institute of Diabetes and Obesity, Helmholtz Center Munich, Neuherberg, Germany.; 6 German Center for Diabetes Research (DZD), Neuherberg, Germany.; 7 Institute of Pharmacology, Medical Faculty, Johannes Kepler University Linz, Linz, Austria.; 8 German Center for Lung Research, Munich, Germany.

**Keywords:** Cell Biology, Beta cells, Insulin, Ion channels

## Abstract

Most overweight individuals do not develop diabetes due to compensatory islet responses to restore glucose homeostasis. Therefore, regulatory pathways that promote β cell compensation are potential targets for treatment of diabetes. The transient receptor potential cation channel subfamily M member 7 protein (TRPM7), harboring a cation channel and a serine/threonine kinase, has been implicated in controlling cell growth and proliferation. Here, we report that selective deletion of *Trpm7* in β cells disrupted insulin secretion and led to progressive glucose intolerance. We indicate that the diminished insulinotropic response in β cell–specific *Trpm7*-knockout mice was caused by decreased insulin production because of impaired enzymatic activity of this protein. Accordingly, high-fat–fed mice with a genetic loss of TRPM7 kinase activity displayed a marked glucose intolerance accompanied by hyperglycemia. These detrimental glucoregulatory effects were engendered by reduced compensatory β cell responses because of mitigated protein kinase B (AKT)/ERK signaling. Collectively, our data identify TRPM7 kinase as a potentially novel regulator of insulin synthesis, β cell dynamics, and glucose homeostasis under obesogenic diet.

## Graphical abstract

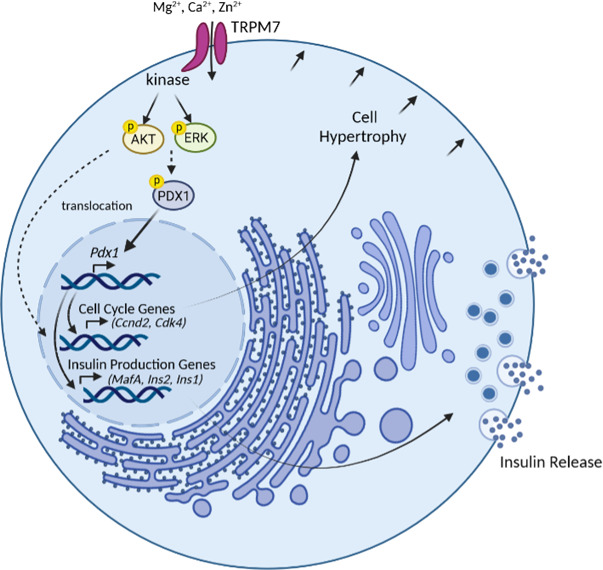

## Introduction

In obese individuals, a combination of environmental and genetic factors may lead to insulin resistance. The onset of insulin resistance is initially offset by enhanced production and secretion of insulin. However, prolonged demand for elevated levels of circulating insulin may eventually result in β cell exhaustion, progressive β cell dysfunction, and development of type 2 diabetes (T2D) (1). Identification of signaling molecules that enhance islet compensatory responses in insulin-resistant states may blaze the trail for novel therapeutic approaches to prevent the progression of insulin resistance to T2D.

Transient receptor potential cation channel subfamily M member 7 (TRPM7) is a ubiquitously expressed membrane protein, consisting of a divalent cation-selective channel linked to a protein kinase domain. The channel moiety of TRPM7 has been implicated in cellular and systemic Mg^2+^ homeostasis (2, 3). Mg^2+^ plays a key role in maintaining β cell health, and while Mg^2+^ deficiency impairs insulin secretion and promotes insulin resistance (4), its supplementation improves β cell function (5). The kinase moiety of TRPM7 belongs to the atypical α-kinase family (6) and has been implicated in controlling numerous cellular processes, such as proliferation, growth, migration, apoptosis, differentiation, and exocytosis (7). α-Kinases are structurally and evolutionarily unrelated to conventional eukaryotic protein kinases, yet they share common sequence motifs and the position of key amino acid residues essential for catalysis (8, 9). The remaining dissimilarities of α-kinases to conventional protein kinases are of potential interest for selective pharmacological targeting (10).

In mice, TRPM7 is a central regulator of embryogenesis and organogenesis, with genetic inactivation of TRPM7 causing early embryonic lethality. It has been suggested that localized increases in the concentration of divalent cations due to transmembrane ion flux through the TRPM7 channel trigger kinase activity engaging signaling pathways that are of fundamental relevance in early development (11). There is strong evidence that increases in TRPM7 activity are required to elicit expression of key cell cycle genes in various cell types (12, 13). In hepatic stellate cells, TRPM7 regulates cell proliferation via phosphatidylinositol-3-kinase (PI3K) and ERK1/2 signaling pathways (14). In lymphocytes, TRPM7 ablation arrests cell proliferation with a high percentage of arrested cells accumulating at the beginning of the cell cycle, suggesting a potential involvement of TRPM7 in processes orchestrating exit from the quiescence/G_0_ phase of the cell cycle (15). Notably, TRPM7 inactivation in pancreatic adenocarcinoma cells decreases proliferation and arrests cells in the G_0_/G_1_ phases of the cell division cycle (16). The kinase moiety of TRPM7 has been suggested to regulate gene transcription through histone modifications. TRPM7 kinase has been reported to be cleaved from the channel domain in a cell type–specific fashion. It would subsequently translocate to the nucleus and bind to components of chromatin-remodeling complexes (17).

Furthermore, TRPM7 plays a role in the regulation of Ca^2+^ signaling in various cell types (18–21). In osteoblasts, the TRPM7 channel modulates cell migration by facilitating Ca^2+^ oscillations (18). TRPM7 has been shown to maintain the Ca^2+^ content of intracellular stores in resting cells. In splenocytes and B lymphocytes, the TRPM7 channel and its kinase moieties regulate store-operated Ca^2+^ entry (SOCE) (19, 20). Dysfunctional SOCE in β cells contributes to the pathogenesis of diabetes and has been reported to disrupt glucose-stimulated Ca^2+^ oscillations in β cells (22).

TRPM7 is highly expressed in human and murine pancreatic β cells (23, 24). Recent studies revealed that TRPM7 contributes to pancreatic endocrine development and β cell proliferation through modulating intracellular Mg^2+^ levels. Furthermore, it has been suggested that TRPM7 channels augment β cell glucose-stimulated Ca^2+^ influx in pancreatic β cells (21). However, it is still not known how changes in TRPM7 kinase–linked activity regulate glucose metabolism and Ca^2+^ signaling in pancreatic β cells.

The present study was designed to clarify the role of TRPM7 in maintaining β cell function under physiological and metabolically challenged conditions. To decipher the function of TRPM7 in glucose homeostasis, we generated a mouse model with a selective deletion of *Trpm7* in β cells (*βTrpm7*-KO mice). Initially, within 4 weeks, these mice exhibited no overt changes in glucose metabolism following recombination. However, a latent induction of glucose intolerance was evident over 28 weeks, suggesting that TRPM7 disruption leads to progressive β cell dysfunction. In-depth metabolic analysis of TRPM7 kinase–dead mice (*Trpm7tm1.1Mkma* *C56BL/6*; herein *Trpm7^R/R^* mice) demonstrated that the kinase moiety of TRPM7 is the key player in this scenario. Specifically, our data identify TRPM7 kinase as a crucial cellular component involved in the preservation of glucometabolic islet function under conditions of diet-induced obesity.

## Results

### Trpm7 deletion in β cells impairs insulin secretion and glucose metabolism.

**Figure 1:**
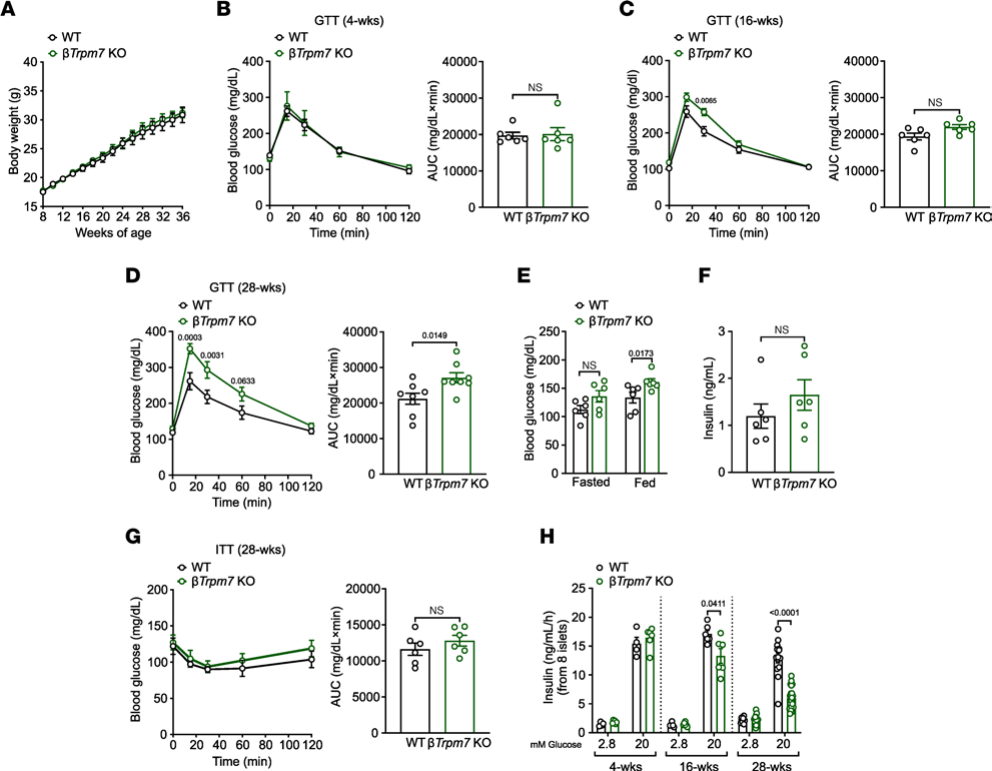
Tissue-specific TRPM7 deletion in β cells impairs glucose homeostasis and glucose-induced insulin secretion. (**A**) Body weight development for 36 weeks (*n* = 6 mice per genotype) monitored in male *βTrpm7*-KO and control littermate mice on chow diet. (**B**–**D**) For glucose tolerance test (GTT) mice were fasted overnight (*n* = 6 mice per genotype). Blood glucose levels (mg/dL) before and within 2 hours after i.p. injection of glucose (2 g/kg of body weight) in wild-type (WT) and *βTrpm7*-KO mice (left panels) and area under the curves (AUC in mg/dL × min; right panels) 4 (**B**), 16 (**C**), and 28 weeks (**D**) postrecombination. (**E**) Blood glucose (mg/dL) in freely fed (*n* = 6 per genotype) or fasted (*n* = 6 mice per genotype) and (**F**) plasma insulin levels (ng/mL) in freely fed (*n* = 6 mice per genotype) were measured in 36-week-old *βTrpm7*-KO and control littermate mice. (**G**) For insulin tolerance test (ITT) mice were fasted for 4 hours at the onset of the light cycle (*n* = 6 mice per genotype). Blood glucose levels (mg/dL) before and within 2 hours after i.p. injection of insulin (0.75 U/kg of body weight) in WT and *βTrpm7*-KO mice (left) and AUC (mg/dL × min; right). (**H**) Insulin secretion (ng/mL/h/8 islets) in isolated islets of male *βTrpm7*-KO and control littermate mice 4, 16, and 28 weeks postrecombination. Islets were incubated for 1 hour in the presence of low glucose and high glucose (*n* ≥ 3 mice per genotype, measured in duplicate). Data show means ± SEM, and statistical differences were assessed by 2-way ANOVA (**B** left–**D** left, and **G** left) or unpaired 2-tailed Student’s *t* test (**B** right–**D** right, **E**, **F**, **G**, right, **H**). Circles in bar graphs represent single values. *P* values are shown above the bars.

To assess the requirement of β cell TRPM7 for insulin secretion and glucose homeostasis, we generated tamoxifen-inducible β cell–specific *Trpm7*-KO mice (*βTrpm7*-KO mice) by crossing *Trpm7^fl/fl^* with MIP-Cre/ERT mice. Pancreatic islets isolated from *βTrpm7*-KO mice revealed efficient β cell–targeted recombination after tamoxifen administration. Notably, no Cre-mediated recombination was detected in any region of the brain or liver in *βTrpm7*-KO mice (Supplemental Figure 1; supplemental material available online with this article; https://doi.org/10.1172/jci.insight.163397DS1). We monitored the metabolic phenotype of *βTrpm7*-KO mice on regular chow diet within 28 weeks. While wild-type and *βTrpm7*-KO mice had similar body weight (Figure 1A), the transgenic mice exhibited progressive glucose intolerance starting 16 weeks postrecombination (Figure 1, B–D). Moreover, *βTrpm7*-KO mice displayed elevated fed blood glucose relative to control littermates after 28 weeks of tamoxifen-induced recombination (Figure 1E). Plasma insulin levels (Figure 1F) and insulin sensitivity (Figure 1G) remained unchanged in both genotypes. Glucose-induced insulin secretion (GIIS) was severely diminished in isolated islets from *βTrpm7*-KO mice after 28 weeks of tamoxifen-induced recombination. Augmentation of glucose concentrations from 2.8 to 20 mM increased insulin release about 5-fold in WT islets, whereas only a 2.5-fold increase of basal insulin exocytosis was observed in the *βTrpm7*-KO islets (Figure 1H). Collectively, these data show that tissue-specific TRPM7 disruption in β cells progressively impairs glucose metabolism, which we hypothesized to be attributable to loss of β cell identity and impaired cell cycle regulation.

### TRPM7 kinase disruption reduces glucose tolerance and GIIS.

**Figure 2:**
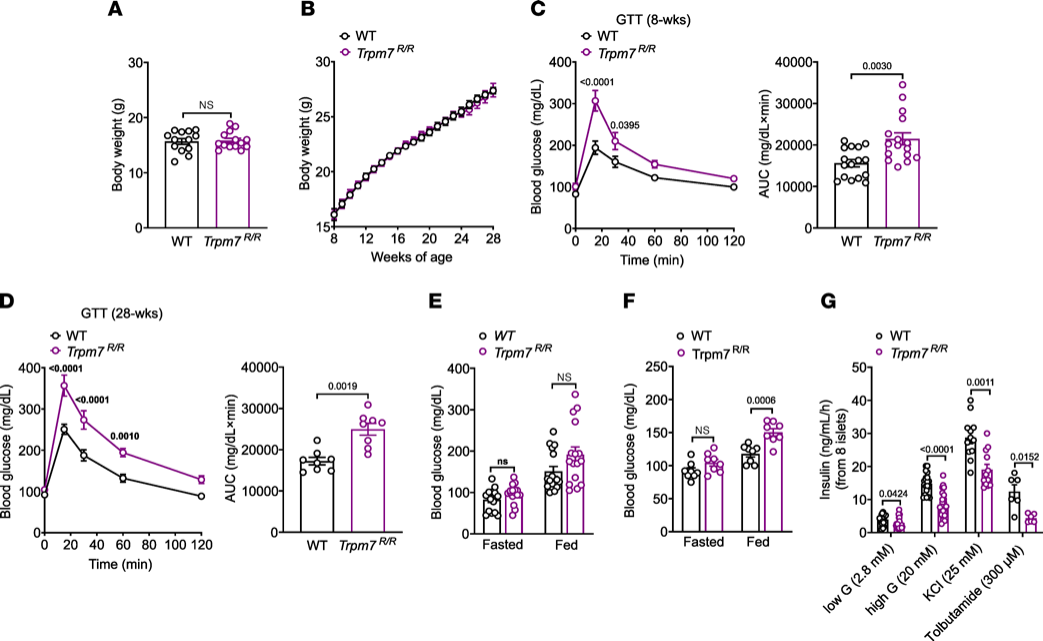
TRPM7 kinase disruption impairs glucose homeostasis and GIIS. (**A** and **B**) Body weight at 8–9 weeks (*n* ≥ 14 mice per genotype) (**A**) and its development for 28 weeks (*n* = 10 mice per genotype) (**B**) monitored in male and female *Trpm7^R/R^* and control littermate mice on chow diet. (**C** and **D**) Blood glucose levels (mg/dL) before and within 2 hours after i.p. injection of glucose (2 g/kg of body weight) and in WT and *Trpm7^R/R^* mice (left panels) and area under the curve (AUC in mg/dL × min; right panels) at age 8–9 weeks (**C**) and 28 weeks (**D**). For glucose tolerance test (GTT) mice were fasted overnight (*n* = 16 mice per genotype (*n* ≥ 8 mice per genotype). (**E** and **F**) Blood glucose (mg/dL) in freely fed (*n* ≥ 8 per genotype) or fasted (*n* ≥ 8 mice per genotype) were measured in *Trpm7^R/R^* and control littermate mice at age 8–9 weeks (**E**) and 28 weeks (**F**). (**G**) Insulin secretion (ng/mL/h/8 islets) in isolated islets of male and female *Trpm7^R/R^* and control littermate mice at 8 weeks of age. Islets were incubated for 1 hour in the presence of low glucose (2.8 mM), high glucose (20 mM), 25 mM KCl or 300 μM tolbutamide (*n* ≥ 3 mice per genotype, measured in duplicate). Data show means ± SEM, and statistical differences were assessed by unpaired 2-tailed Student’s *t* test (**A**, **C** right, **D** right, **E**–**G**) or 2-way ANOVA (**C** left, **D** left). Circles in bar graphs represent single values. *P* values are shown above the bars.

To decipher whether the impaired glucose metabolism is linked to the TRPM7 channel or kinase moiety, we took advantage of a mouse model harboring a single point mutation at the active site of the enzyme (*Trpm7^R/R^*). We monitored the metabolic phenotype of *Trpm7^R/R^* mice within 28 weeks. When fed a standard chow diet, *Trpm7^R/R^* mice showed no difference in body weight compared to their WT controls (Figure 2, A and B). However, *Trpm7^R/R^* mice displayed glucose intolerance at 8 weeks of age that became more prominent in 28-week-old mice (Figure 2, C and D, and Supplemental Figure 2A). Although the concentrations of blood glucose remained unchanged in fasted and fed 8-week-old *Trpm7^R/R^* mice relative to controls (Figure 2E), an elevated fed blood glucose was detected in *Trpm7^R/R^* mice at 28 weeks of age (Figure 2F). Insulin sensitivity and plasma insulin levels remained unaffected within this period (Supplemental Figure 2, B–E). This observation pointed to a putative role of the TRPM7 kinase in sustaining islet function and modulating insulin secretion. To test this hypothesis, we investigated GIIS using isolated islets from *Trpm7^R/R^* mice and control littermates. In accord with the impaired glucose tolerance observed in vivo, TRPM7 kinase disruption reduced maximal insulin secretion capacity in isolated islets (Figure 2G and Supplemental Figure 2F). Augmenting the glucose concentration from 2.8 to 20 mM enhanced insulin exocytosis about 4-fold in WT islets, whereas insulin exocytosis only rose 3-fold in isolated islets from 8-week-old *Trpm7^R/R^* mice (Figure 2G) and 2.2-fold at 28 weeks of age (Supplemental Figure 2F). Membrane depolarization of β cells by exposure to either 25 mM KCl or 300 μM tolbutamide increased insulin secretion to about 7- and 3.5-fold, respectively, while insulin exocytosis only increased 6.5- and 2-fold in isolated islets of *Trpm7^R/R^* mice at 8 weeks of age. Interestingly, basal insulin secretion was significantly lower in *Trpm7^R/R^* islets relative to WT controls (Figure 2G and Supplemental Figure 2F).

### TRPM7 kinase inactivation has no effect on glucose-induced Ca^2+^ responses and TRPM7-mediated ion currents.

**Figure 3:**
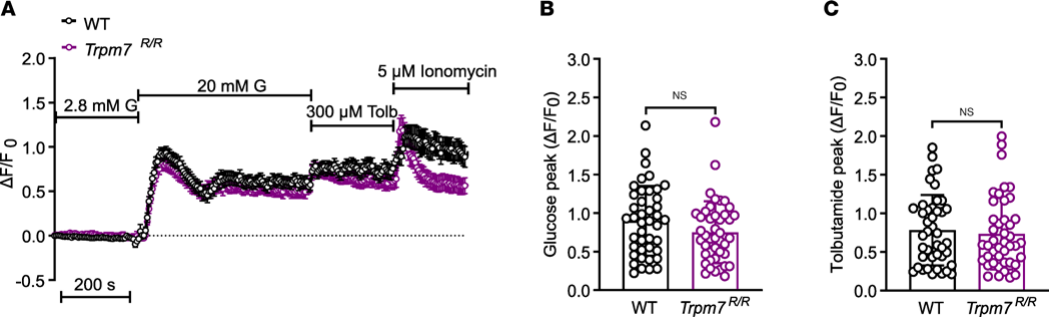
TRPM7 kinase disruption has no effect on glucose-induced Ca^2+^ responses. (**A**) Intact WT (*n* = 42 cells, from 3 mice) and *Trpm7^R/R^* (*n* = 43, from 3 mice) islets were loaded with 4 μM fluo-4 AM, and alterations in [Ca^2+^]_i_ of individual cells were monitored by confocal microscopy after increasing the extracellular glucose concentration from 2.8 to 20 mM and applying 300 μM tolbutamide. Ionomycin (5 μM) was used as a positive control. (**B** and **C**) Average of Ca^2+^ influx peaks assessed from baseline after glucose (**B**) and tolbutamide (**C**) stimulation in WT and *Trpm7^R/R^* β cells. The cells that displayed no increase in [Ca^2+^]_i_ in response to high glucose concentration are excluded from the results. Data are given as mean ± SEM (circles in bar graphs represent single values), and statistical differences were assessed by unpaired 2-tailed Student’s *t* test (**B** and **C**).

β Cells display a characteristic Ca^2+^ oscillation pattern in response to high glucose concentration, thus regulating the exocytosis of insulin. Hence, we next asked whether impaired Ca^2+^ responses are attributable to declines in glucose-induced rises in insulin secretion in *Trpm7^R/R^* islets. Exposure to glucose initially induced a rapid increase in [Ca^2+^]_i_ followed by continuous oscillations in a subset of WT islet cells. *Trpm7^R/R^* islets responded similarly to 20 mM glucose in terms of [Ca^2+^]_i_ transients when compared to WT islets (Figure 3, A and B). The responses to tolbutamide (Figure 3, A and C) and KCl (Supplemental Figure 3, A and B) under identical conditions were also very similar to one another, supporting that canonical K_ATP_ signaling is not affected by loss of TRPM7 kinase function. Neither the Ca^2+^ oscillation frequency nor the average amplitude of the oscillatory response was different from one another in both genotypes (Supplemental Figure 4, A–C). Taken together, these data demonstrate that TRPM7 kinase disruption does not affect glucose-induced Ca^2+^ responses in β cells.

**Figure 4:**
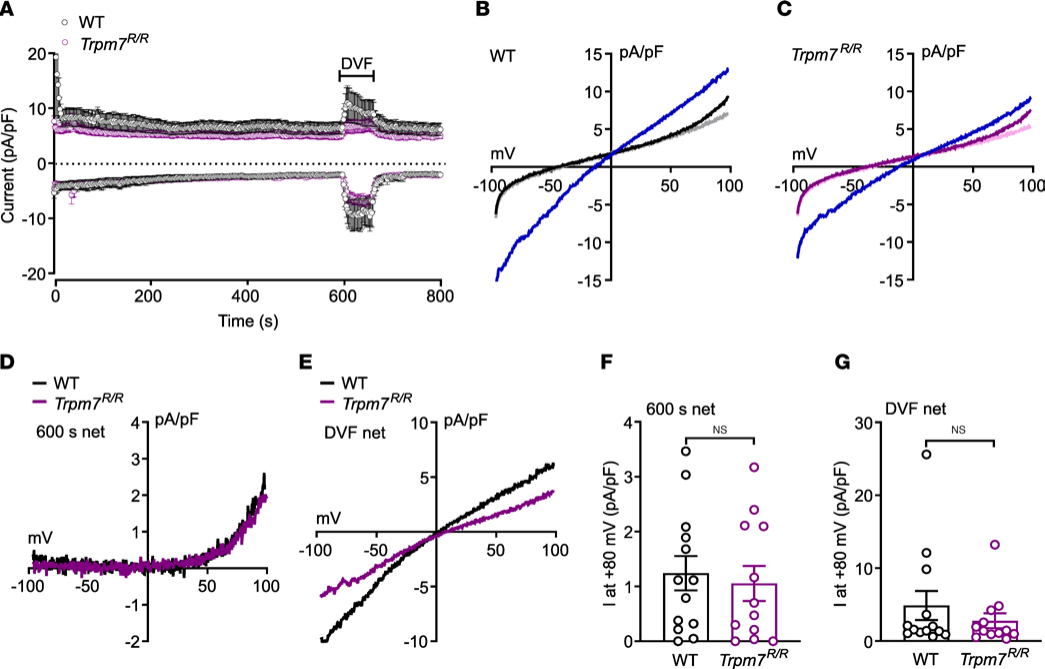
TRPM7 kinase inactivation has no effect on TRPM7 current activity. Whole-cell currents recorded from islet cells of WT (**A**, **D**, **E**, black trace, **B**) and *Trpm7^R/R^* mice (**A**, **D**, **E**, purple trace, **C**), using Mg^2+^-free pipette solution (buffered by 10 mM EDTA). (**A**) In- and outward current amplitudes at –80 mV (lower traces) and +80 mV (upper traces), extracted from whole-cell currents mediated by voltage ramps, applied at 0.5 Hz, spanning from –100 mV to 100 mV within 50 ms, in the absence of intracellular Mg^2+^ in WT and *Trpm7^R/R^* islet cells, plotted versus time. Divalent-free solution (DVF, buffered by EDTA) was applied from 600 to 660 seconds (bar). (**B** and **C**) Current-voltage relationships (IVs) of the minimal basic current (gray, light purple), the current at 600 seconds (black, purple, right before DVF) and in DVF solution (blue) in WT (**B**) and in *Trpm7^R/R^* islet cells (**C**). (**D** and **E**) IVs of the net current at 600 seconds (600 s net = current at 600 seconds minus basic current) and in DVF solution (DVF net = current in DVF minus basic current) in WT (black) and *Trpm7^R/R^* islet cells (purple). (**F** and **G**) Summary of the net current amplitudes at +80 mV from IVs at 600 seconds (600 s net; **F**) and in DVF solution (DVF net; **G**) in cells isolated from WT (black) and *Trpm7^R/R^* mice (purple). All currents were normalized to the cell capacitance (pA/pF). Data are plotted as means ± SEM (**A**, **F**, and **G**) or means (**B**–**E**). Data are from 13 cells for WT and 12 cells for *Trpm7^R/R^*. Data are given as means ± SEM (circles in bar graphs represent single values), and statistical differences were assessed by unpaired 2-tailed Student’s *t* test (**F** and **G**).

As TRPM7 channels are activated by depletion of intracellular Mg^2+^ (2), we determined the effects of intracellular Mg^2+^ removal on the underlying whole-cell currents in pancreatic islet cells isolated from WT and *Trpm7^R/R^* mice (Figure 4, A–G). Upon break-in by the patch pipette, most islet cells revealed huge outward currents, probably mediated by a voltage-dependent potassium efflux, which rapidly vanished after infusion of the cesium-based Mg^2+^-free pipette solution and washout of the cytosolic potassium content (Figure 4A). However, within 600 seconds, small currents with a current-voltage relationship reminiscent of TRPM7 activation developed in both the WT (Figure 4B, black trace) and *Trpm7^R/R^* (Figure 4C, purple trace) islet cells. To verify the dependency of these small outward currents on TRPM7 activation, we applied DVF (buffered by EDTA), resulting in substantially increased in- and outward currents in islet cells of both genotypes as a hallmark of TRPM7 activation (Figure 4, A–C). Since removal of external divalent cations increases TRPM7’s permeability to monovalent cations, both in- and outward currents increased and the current-voltage relationship switched from slightly outward rectification to a nearly linear shape (Figure 4, B and C, blue traces). Figure 4, D and E, show the current-voltage relations of the basal current–subtracted net TRPM7 current, extracted after 600 seconds (Figure 4D) and the basal current–subtracted net current measured in DVFs (Figure 4E) in islet cells from WT (black traces) and *Trpm7^R/R^* mice (purple). The amplitudes of net TRPM7 currents induced by depletion of free intracellular Mg^2+^ at +80 mV in the presence (Figure 4, D and F) and absence of extracellular divalent cations (Figure 4, E and G) in islet cells isolated from WT and *Trpm7^R/R^* mice were not significantly different, suggesting that in islet cells TRPM7-induced currents are not affected by the presence or absence of its kinase activity.

### Genetic loss of TRPM7 kinase does not alter the pancreatic islet cytoarchitecture.

**Figure 5:**
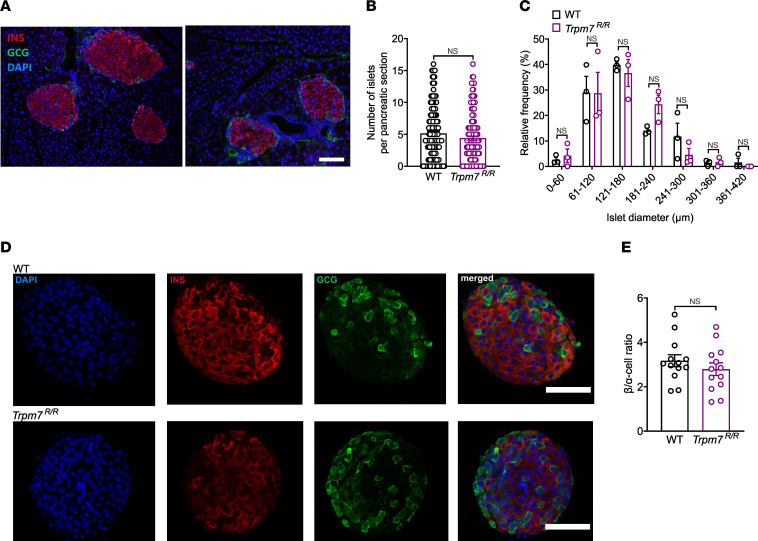
Morphology of WT and *Trpm7^R/R^* pancreatic islets. (**A**) Immunofluorescent insulin (INS, red) and glucagon (GCG, green) staining of pancreatic cryosections of WT and *Trpm7^R/R^* mice. Nuclei were stained with DAPI (blue) and scale bars represent 100 μm. (**B**) Number of islets per pancreatic cryosection (*n* = 140 slides, 3 mice per genotype) and (**C**) relative frequency plot of islet diameter comparing WT with *Trpm7^R/R^* islets (*n* = 140 slides, 3 mice per genotype). (**D**) Confocal images of WT and *Trpm7^R/R^* islets stained for insulin (β cells, red) and glucagon (α cells, green). Nuclei were stained with DAPI (blue) and scale bars represent 100 μm. (**E**) Quantification of the ratio of the number of β and α cells per pancreatic islet in WT and *Trpm7^R/R^* mice (*n* = 13, 3 mice per genotype). Data are given as means ± SEM (circles in bar graphs represent single values), and statistical differences were assessed by Mann-Whitney test (**B**) and unpaired 2-tailed Student’s *t* test (**C** and **E**).

Next, we asked whether loss of TRPM7 kinase function affects pancreatic islet development. We observed that islet density (Figure 5, A and B) as well as islet size distribution (Figure 5C) were similar in both genotypes. Islet size ranged between >45 and <420 μm in both genotypes, with the highest frequency found between 61 and 180 μm (Figure 5C). In both genotypes, approximately 60%–70% of the endocrine cells within the islets were insulin-positive β cells, localized in the central part of the islets, while glucagon-positive α cells were located at the islet periphery surrounding β cells (Figure 5, A and D). Moreover, the ratio of β to α cells remained unchanged in both genotypes (Figure 5E).

### TRPM7 kinase disruption attenuates insulin biosynthesis.

**Figure 6:**
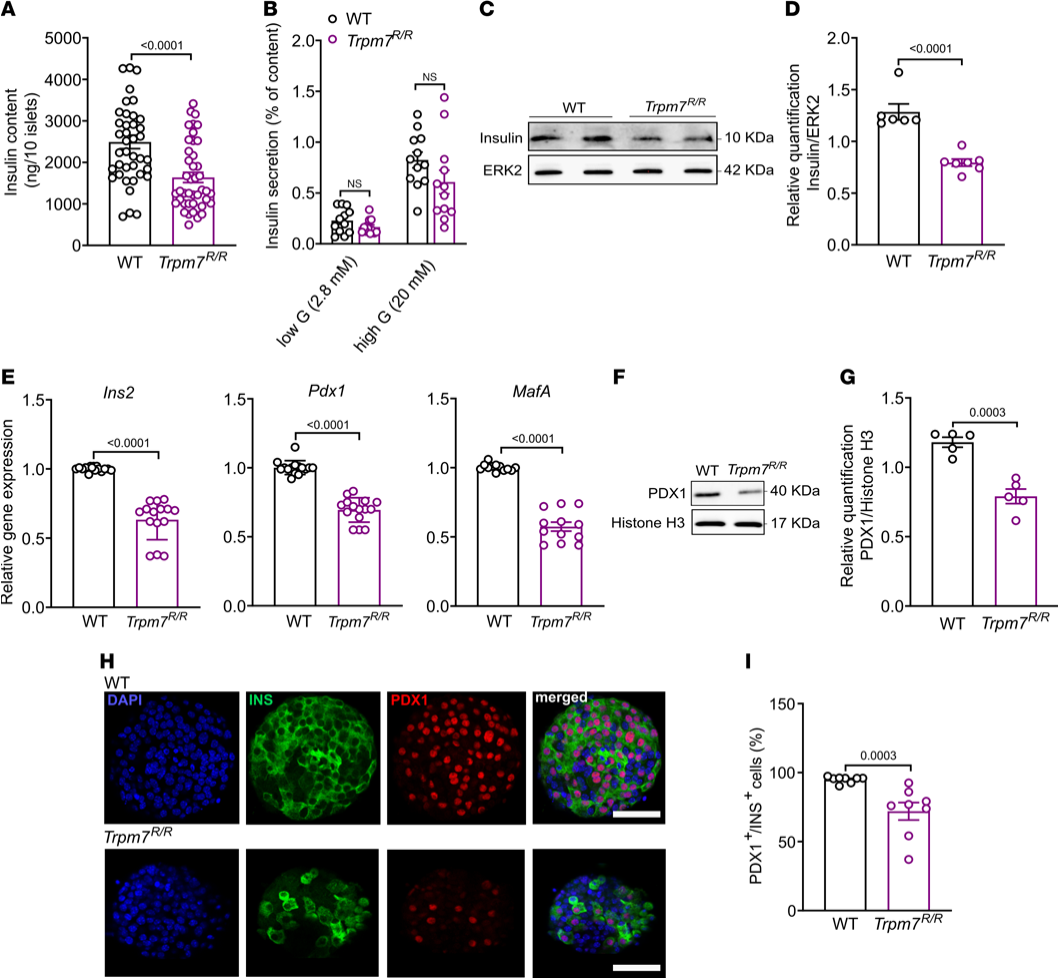
TRPM7 kinase disruption impairs insulin production. (**A**) Total insulin content of pooled WT versus *Trpm7^R/R^* islets. At least 40 groups of 10 size-matched WT and *Trpm7^R/R^* islets were compared. (**B**) Percentage of insulin content secreted from intact WT or *Trpm7^R/R^* islets after incubation with either low glucose (2.8 mM) or high glucose (20 mM) (*n* = 6 mice per genotype, measured in duplicate). (**C** and **D**) Western blot detection of the insulin in lysates of purified islets from WT and *Trpm7^R/R^* mice (*n* ≥ 5, 4 mice per genotype). Insulin was normalized to ERK2 as loading control. (**E**) Expression levels of *Ins2*, *Pdx1*, and *MafA* analyzed by qRT-PCR from RNAs isolated from pancreatic islet from WT and *Trpm7^R/R^* mice. (**F** and **G**) Western blot detection of the PDX1 in lysates of purified islets from WT and *Trpm7^R/R^* mice (*n* = 4, 4 mice per genotype). PDX1 was normalized to histone H3 as loading control. (**H**) Confocal images of WT and *Trpm7^R/R^* islets stained for DAPI (blue), insulin (green), PDX1 (red). The scale bar represents 100 μm. (**I**) Percentage of PDX1-positive cells from the population (100%) of insulin-positive cells per pancreatic islet in WT and *Trpm7^R/R^* mice (*n* = 8, 4 mice per genotype). Data are given as means ± SEM (circles in bar graphs represent single values), and statistical differences were assessed by Mann-Whitney test (**A**) or unpaired 2-tailed Student’s *t* test (**B**, **D**, **E**, **G**, and **I**). *P* values are shown above the bars.

Next, we measured the insulin content in islets of both genotypes and noted a remarkable decrease of the insulin content in *Trpm7^R/R^* islets relative to WT controls (Figure 6A). While GIIS was also decreased in *Trpm7^R/R^* islets (see Figure 2G), the GIIS normalized to the content was not significantly different between WT and *Trpm7^R/R^*. These results indicate that the TRPM7 kinase plays a role in insulin biosynthesis rather than insulin release (Figure 6B). Western blot analysis supported the declines in insulin levels (Figure 6, C and D). To dissect the cellular mechanism underlying the reduced insulin content of *Trpm7^R/R^* islets, we performed real-time quantitative reverse transcription PCR (qRT-PCR) analyses and identified reduced expression of key genes involved in insulin production in *Trpm7^R/R^* islets relative to those in WT cells, including *Ins2*, pancreatic and duodenal homeobox 1 (*Pdx1*), and V-maf musculoaponeurotic fibrosarcoma oncogene homolog A (*MafA*) (Figure 6E). The reduced expression of PDX1 protein in *Trpm7^R/R^* islets was supported by Western blotting and immunohistochemistry (Figure 6, F–I). In addition, we demonstrated a remarkable decrease in insulin content as well as expression levels of PDX1 protein in isolated islets from *βTrpm7* KO after 28 weeks of tamoxifen-induced recombination (Supplemental Figure 5, A–C). These data suggest that the reduced insulin content of *Trpm7^R/R^* and *βTrpm7*-KO islets may be caused by decreased insulin synthesis secondary to a reduction of *Pdx1* expression.

### TRPM7 kinase inactivation impairs HFD-induced β cell mass expansion.

**Figure 7:**
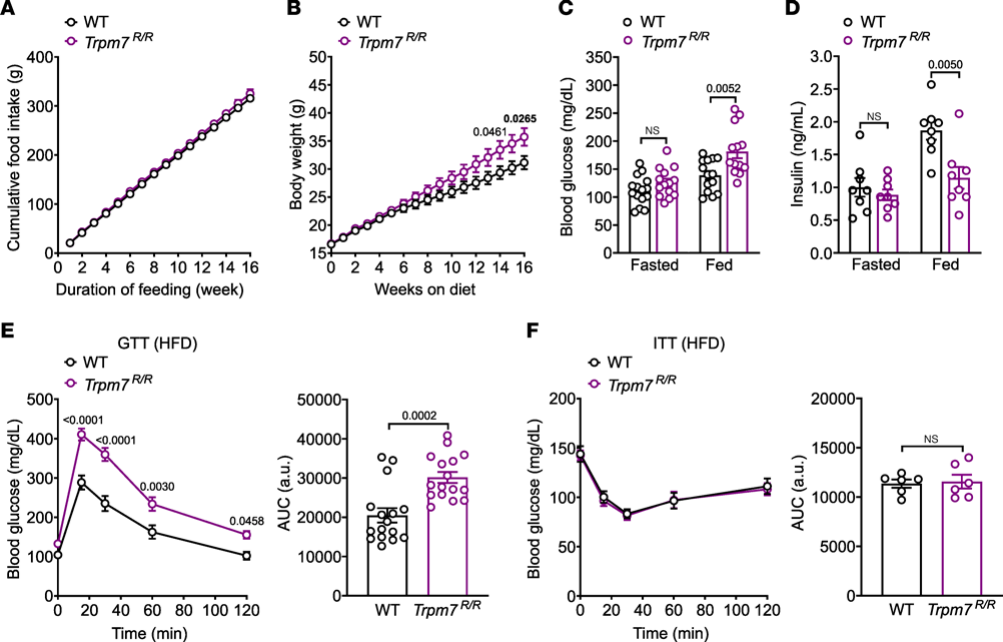
TRPM7 kinase disruption impairs glucose homeostasis in obese mice. Adult mice (*Trpm7^R/R^* and control littermates) maintained on an HFD for 16 weeks. (**A**) Cumulative food intake (*n* ≥ 6 mice per genotype), (**B**) body weight (*n* ≥ 14 mice per genotype), (**C**) blood glucose (mg/dL) in freely fed (*n* ≥ 14 mice per genotype) or fasted (*n* ≥ 12 mice per genotype), and (**D**) plasma insulin levels (ng/mL) in freely fed (*n* = 8 mice per genotype) or fasted (16 hours overnight) (*n* = 8 mice per genotype) in male and female *Trpm7^R/R^* and control littermate mice were measured. (**E** and **F**) Blood glucose levels (mg/dL) before and within 2 hours after i.p. injection of (**E**) glucose (2 g/kg of body weight) and (**F**) insulin (0.75 U/kg of body weight) in WT and *Trpm7^R/R^* mice (left panels) and area under the curves (AUC in mg/dL × min; right panels). For glucose tolerance test (GTT; **E**) mice were fasted overnight (*n* ≥ 20 mice per genotype) and for insulin tolerance test (ITT; **F**) mice were fasted for 4 hours at the onset of the light cycle (*n* ≥ 14 mice per genotype). Data show means ± SEM and statistical differences were assessed by unpaired 2-tailed Student’s *t* test (**C**, **D**, **E** right, and **F** right) or 2-way ANOVA (**E** and **F**, left panels). Circles in bar graphs represent single values. *P* values are shown above the bars.

**Figure 8:**
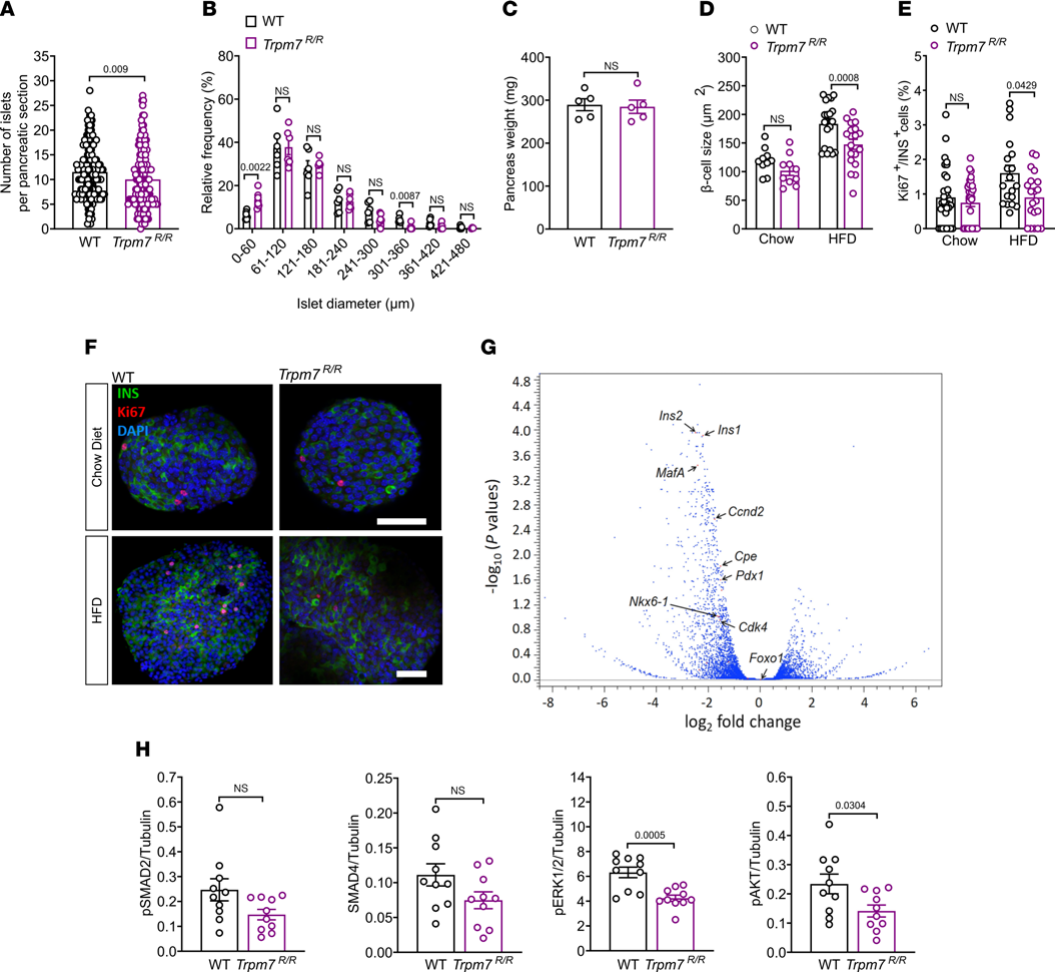
TRPM7 kinase disruption reduces compensatory β cell responses due to a mitigated AKT/ERK signaling. (**A**) Number of islets per pancreatic cryosection (*n* = 144 slides, 3 mice per genotype), (**B**) relative frequency plot of islet diameter comparing WT with *Trpm7^R/R^* islets (*n* = 144 slides, 6 mice per genotype). (**C**) Pancreas weight of WT and *Trpm7^R/R^* mice maintained on an HFD for approximately 16 weeks (*n* = 5 mice per genotype). (**D**) β Cell size (≥10 islets, at least 3 mice per genotype) and (**E**) percentage of Ki67-positive cells from the population (100%) of the insulin-positive cells per pancreatic islet in WT and *Trpm7^R/R^* mice under the chow or HFD for 16 weeks (*n* = 20, 5 mice per genotype). (**F**) Confocal images of WT and *Trpm7^R/R^* islets stained for DAPI (blue), insulin (green), and Ki67 (red). The scale bar represents 100 μm. (**G**) For RNA-Seq analysis, islet RNA was collected from *Trpm7^R/R^* mice and the control littermates that had been maintained on an HFD for approximately 16 weeks (*n* = 3 mice per genotype, age: ~24 weeks). Volcano plot with downregulated and upregulated genes. Differentially expressed genes (DEGs) were identified (*P* < 0.05) by using EdgeR method. DEGs are expressed as log_2_ fold change over control with an adjusted *P* value for each gene. (**H**) Assessment of the activity of the cell signaling molecules SMAD2, SMAD4, ERK1/2, and AKT using Bio-Plex assay and phospho-specific antibodies on lysates of isolated islets from WT and *Trpm7^R/R^* mice (*n* = 10, measured in duplicates, 10 mice per genotype) under 16 weeks of HFD. Data are normalized to Tubulin content. Data show means ± SEM and statistical differences were assessed by Mann-Whitney test (**A**) or unpaired 2-tailed Student’s *t* test (**B**–**E**, and **H**). Circles in bar graphs represent single values. *P* values are shown above the bars.

*Pdx1* is implicated in compensatory β cell mass expansion in response to diet-induced insulin resistance (25). Therefore, we examined whether TRPM7 kinase is required for maintenance of pancreatic islet function under HFD feeding. Although cumulative food intake (Figure 7A) was comparable in both genotypes, *Trpm7^R/R^* mice underwent pronounced increases in body weight and blood glucose levels (Figure 7, B and C) and a significant reduction in plasma insulin in the fed state relative to WT littermates (Figure 7D). Of note, during HFD feeding, glucose tolerance was severely impaired in *Trpm7^R/R^* mice relative to WT littermates (Figure 7E). Both groups of mice showed similar reductions in blood glucose levels in an insulin tolerance test (Figure 7F). Interestingly, the number of islets per pancreatic section (Figure 8A) as well as the frequency distribution of larger (301–360 μm) islets were reduced, while the distribution of small (0–60 μm) islets was increased in *Trpm7^R/R^* pancreatic slices (Figure 8B). However, total pancreatic weight did not differ between high-fat–fed *Trpm7^R/R^* and control littermates (Figure 8C). As normal islet compensation involves an expansion of β cell mass achieved by β cell hypertrophy and proliferation (26, 27), we examined β cell size, proliferation, and survival rates in response to HFD. Notably, *Trpm7^R/R^* islets displayed a significant decrease in β cell size relative to control littermates (Figure 8D). Ki67 was used as a proliferation marker. The abundance of Ki67-positive β cells was markedly reduced in *Trpm7^R/R^* islets compared with control littermates (Figure 8, E and F). Thus, these results suggest that loss of TRPM7 kinase restrains β cell proliferation in response to HFD. Importantly, we did not detect any TUNEL-positive, i.e., apoptotic, β cells in WT and *Trpm7^R/R^* islets (Supplemental Figure 6A). Furthermore, to investigate whether reduced β cell proliferation in *Trpm7^R/R^* mice is associated with impaired SOCE, we monitored passive Ca^2+^ release in response to the sarcoplasmic/endoplasmic reticulum calcium ATPase (SERCA) pump blocker cyclopiazonic acid followed by SOCE after Ca^2+^ re-addition. However, there was no significant difference both in passive Ca^2+^ release and in subsequent SOCE between β cells derived from *Trpm7^R/R^* and WT genotypes (Supplemental Figure 6, B–D).

### TRPM7 kinase inactivation reduces expression levels of genes involved in insulin production and cell cycle regulation.

We investigated the gene expression profile by RNA sequencing using RNA prepared from isolated islets of high-fat–fed *Trpm7^R/R^* and WT mice. We identified upregulation of 382 genes and downregulation of 1,615 genes in *Trpm7^R/R^* islets. Interestingly, genes critical to insulin production were found to be downregulated, including *Ins2*, *MafA*, *Pdx1*, *Cpe*, and *Nkx6-1*. We also observed downmodulation of genes involved in cell cycle regulation, such as cyclin-dependent kinase 4 (*Cdk4*) and *Ccnd2*, and several proliferation markers, including *Cirp*, *Mll5*, and *Pimerg* (Supplemental Table 1). No changes were observed in the expression levels of genes involved in glucose sensing and exocytosis (Supplemental Table 2). A volcano plot (Figure 8G) and heatmaps (Supplemental Figure 7, A and B) illustrate the differential expression of genes involved in insulin biosynthesis, cell cycle progression, and cellular proliferation. A summary of the genes involved in insulin production and cell cycle regulation that were downregulated in islets lacking TRPM7 kinase is shown in Supplemental Figure 7, C and D.

### TRPM7 regulates β cell function via protein kinase B (AKT) and ERK1/2 signaling pathways.

TRPM7 kinase regulates TGF-β/SMAD signaling (28). This signaling pathway has been implicated in various cellular processes, including proliferation, differentiation, apoptosis, and cell migration (29). To gain insight into the connection between TRPM7 kinase and signaling cascades triggering compensatory islet responses to an HFD, we employed a bead-based Bio-Plex assay to simultaneously measure the phosphorylation status of multiple TGF-β/SMAD signaling proteins in the same sample. We examined the phosphorylation status of SMAD2 (Ser465/Ser467), SMAD3 (Ser423/Ser425), AKT (Ser473), and ERK1/2 (Thr185/Tyr187) as well as total protein levels of SMAD4 in isolated islets from *Trpm7^R/R^* and WT mice on an HFD for 16 weeks. A slight decrease was detected in the phosphorylation levels of SMAD2 and total SMAD4. However, the differences did not reach statistical significance. *Trpm7^R/R^* mice displayed a significant reduction of ERK-dependent signaling under HFD feeding. Interestingly, our results demonstrated a 40% reduction of AKT phosphorylation in *Trpm7^R/R^* mice fed with an HFD (Figure 8H). Quantification of SMAD3 phosphorylation status was below the detection limit in both genotypes and excluded from the study.

Next, we asked whether reduced ERK1/2 and AKT phosphorylation in *Trpm7^R/R^* islets is attributable to the dysfunctional TRPM7 in β cells. To address this question, we measured the phosphorylation status of ERK1/2 and AKT in isolated islets from *βTrpm7*-KO mice and control littermates on 16 weeks of an HFD. Notably, *βTrpm7*-KO mice demonstrated a significant decrease in both ERK- and AKT-dependent signaling (Supplemental Figure 8, A and B), supporting the role of TRPM7 in modulating these signaling cascades in β cells.

### TRPM7 overexpression induces GIIS in MIN6 cells.

To test whether enhanced expression of *Trpm7* in β cells induces insulin secretion, we used cultured MIN6 mouse insulinoma cells as an in vitro model. We transiently transfected MIN6 cells with *Trpm7* WT or *Trpm7^R/R^* plasmids or with empty vector (Supplemental Figure 9A). The MIN6 cells treated with the *Trpm7^R/R^* plasmid had a GIIS response similar in magnitude to that of MIN6 cells treated with an empty vector. In contrast, MIN6 cells transfected with the *Trpm7* WT plasmid displayed a pronounced increase in GIIS (Supplemental Figure 9B). This response pattern is in good agreement with the data obtained with isolated islets from *βTrpm7*-KO and *Trpm7^R/R^* mice. Next, we investigated the effect of *Trpm7* overexpression in MIN6 cells on the expression levels of key signaling proteins. Western blotting studies showed that treatment of MIN6 cells with *Trpm7* WT plasmid led to a significant increase in phosphorylated forms of ERK1/2 and AKT (Ser473), as compared with cells treated with empty vectors. The expression levels of total ERK and AKT remained essentially unchanged after *Trpm7* WT overexpression (Supplemental Figure 9, C–E).

## Discussion

We report here that TRPM7 regulates glucose homeostasis and compensatory pancreatic islet responses via its kinase moiety. Impaired glucose tolerance and GIIS were comparable among *βTrpm7*-KO and *Trpm7^R/R^* genotypes. Importantly, mice from both genotypes developed age-dependent rises in dysfunctional glucose metabolism and declines in GIIS. Reduced insulin secretion in response to various insulin secretagogues in *Trpm7^R/R^* suggests a salient role of this α-kinase in maintaining β cell function. Intracellular Ca^2+^ transients are the final trigger for insulin exocytosis. Studies with *Trpm7^R/R^* pancreatic islets showed that glucose and high KCl-induced Ca^2+^ responses remained unaffected, suggesting that the reduction of GIIS in *Trpm7^R/R^* mice is not caused by impaired Ca^2+^ signaling in β cells. In addition, TRPM7-like currents in *Trpm7^R/R^* islets were comparable to those of WT cells, demonstrating that the channel moiety of TRPM7 remains intact in this mouse model. Interestingly, we found a pronounced reduction of insulin content accompanied by reduced *Pdx1* transcript and protein levels in *Trpm7^R/R^* and *βTrpm7*-KO mice. In humans, mutations of *Pdx1* are strongly associated with diabetes (30). Previous studies have demonstrated a correlation between low PDX1 levels and β cell dysfunction (31, 32), because PDX1 directly regulates the expression of the insulin gene and other components of the GIIS pathway, including *MafA* (33, 34). Therefore, we suggest that *Pdx1* downregulation might suppress insulin production in *Trpm7^R/R^* and *βTrpm7*-KO mice.

Importantly, *Pdx1* and *MafA* are the key β cell markers and major transcription factors to maintain β cell identity. Altered identity of β cells has been proposed as an underlying mechanism of diabetes progression in patients (35, 36). Furthermore, previous studies linked overexpression of PDX1 to the upregulation of several cell cycle genes and increases in β cell proliferation (37, 38). Therefore, we attribute the age-dependent progressive impairment in glucose metabolism in *Trpm7^R/R^* and *βTrpm7*-KO mice to the gradual loss of β cell identity and reduced β cell proliferation due to *Pdx1* downregulation.

Accumulating evidence has underscored the pivotal role of PDX1 in β cell expansion and survival in response to an HFD challenge (25). Therefore, we set out to define the role of TRPM7 kinase in β cell survival and compensatory hypertrophy in response to an HFD. Thus, an obesogenic diet resulted in increased body weight, hyperglycemia, reduced insulin levels, and glucose intolerance in *Trpm7^R/R^* mice relative to WT controls. These phenotypic changes were not caused by severe insulin resistance in *Trpm7^R/R^* mice, because insulin tolerance was comparable in both genotypes. It is worth mentioning that high-fat–fed *Trpm7^R/R^* mice became more severely hyperglycemic than control littermates after 16 weeks of obesogenic diet, especially in the fed state. Collectively, our data are compatible with the notion that the pronounced impairment of glucose homeostasis in *Trpm7^R/R^* mice is attributable to impaired compensatory β cell mass expansion and proliferation in response to obesogenic diet. Glucose intolerance observed in the *Trpm7^R/R^* mice on the HFD was consistent with the metabolic phenotype of *Pdx1*^+/–^ animals (39). Like our findings in *Trpm7^R/R^* mice, high-fat feeding induced a similar weight gain in *Pdx1*^+/–^ animals relative to WT controls (25). Taken together, we suggest that reduced *Pdx1* expression in *Trpm7^R/R^* mice dampens both insulin production and compensatory β cell mass expansion, entailing compromised glucose tolerance in high-fat–fed *Trpm7^R/R^* mice.

Prior to our work, Altman et al. (21) observed that β cell proliferation induced by 2-week HFD was significantly reduced in TRPM7 deficient β cells. In agreement with our finding, TRPM7 disruption in β cells did not alter glucose tolerance within 4 weeks of recombination. However, the authors suggested that reduced proliferation observed after exposure to obesogenic diet was mediated by impeded Mg^2+^ influx into β cells during proliferation (21). In this context, it is worth mentioning that TRPM7 channel and kinase activities are mutually interdependent, in that the kinase functionality requires the influx of Mg^2+^ through the channel pore (40). Therefore, we put forward an alternative explanation and suggest that hampered β cell proliferation may be attributable to reduced kinase activity that results from declines in Mg^2+^ levels. Furthermore, our histological studies of *Trpm7^R/R^* mice pancreas do not agree with the developmental changes Altman et al. showed after TRPM7 inactivation (21). This difference might point to the role of the channel moiety of TRPM7 in early events influencing pancreatic endocrine development.

RNA-Seq studies with RNA isolated from high-fat–fed *Trpm7^R/R^* and control islets demonstrated that TRPM7 kinase deficiency downregulated the genes involved in insulin biosynthesis, cell cycle progression, and proliferation. Notably, TRPM7 kinase disruption engenders reduced expression of 2 early G1/S phase molecules, cyclin D2 and *Cdk4* in high-fat–fed mice. It has previously been demonstrated that mice lacking *Cdk4* exhibit islet deformity and a reduced size of islets accompanied by diminished insulin production, whereas activation of the CDK4 pathway resulted in β cell hyperplasia (41). Interestingly, reduced pancreas size in *Cdk4*-deficient mice is thought to result from impaired mesenchymal development and decreased numbers of PDX1^+^ pancreatic progenitor cells (42). Moreover, CDK4 enhances β cell replication within adult islets and activates progenitor cells within adult pancreatic ductal epithelium in response to partial pancreatectomy (43). We suggest that reduced islet size, and impaired β cell proliferation in high-fat–fed *Trpm7^R/R^* mice, might be attributable at least partially to reduced CDK4 expression in pancreatic islets. In addition, we noted that the transcript levels of several other proliferation markers, including *Cirp*, *Mll5*, and *Pimerg*, were substantially reduced in high-fat–fed TRPM7 *Trpm7^R/R^* mice. Interestingly, CIRP activation occurs downstream of various stress stimuli and is known to regulate cell survival and cell proliferation, particularly during stress (44).

In mouse models of diet-induced obesity, high-fat feeding has been linked to ER stress in β cells, resulting in the inability to trigger an appropriate unfolded protein response (UPR), potentially leading to β cell apoptosis (45). Previous studies show that *Pdx1*^+/–^ β cells are more susceptible to ER stress under high-fat feeding (25). PDX1 plays a crucial role in the regulation of genes involved in ER function, including disulfide bond formation, protein folding, and the UPR. Here, we show the downregulation of several ER-related genes in *Trpm7^R/R^* islets, including genes encoding enzymes critical for disulfide bond formation in the ER (*Pdia4* and *Pdia6*), ER chaperone (*Hspa5*), and mediators of UPR pathways (*Atf4*), which are direct transcriptional targets of PDX1 (25). Although it has been suggested that *Pdx1* deficiency promotes ER stress–associated cell death, we did not detect apoptosis in WT and *Trpm7^R/R^* islets, even when challenged by high-fat feeding. This observation is fully in line with a recent study by Barella et al., who did not detect any apoptotic β cells in β-*barr1*-KO mice in the presence of severely impaired *Pdx1* expression (46). Furthermore, Altman et al. reported that TRPM7 KO has no effect on β cell apoptosis (21). Previous studies demonstrated that knockdown of *Pdx1* in rat insulinoma cells (INS-1) results in a reduced SERCA2b expression and decreased ER Ca^2+^ levels (47). Importantly, TRPM7 kinase deficiency has been shown to suppress SOCE in T- cells and B lymphocytes (19, 20). Nevertheless, we found that both SOCE and ER Ca^2+^ storage were unaffected in *Trpm7^R/R^* islets (Supplemental Figure 6, B and C), ruling out a major impediment of this pathway in pancreatic β cells from *Trpm7^R/R^* mice.

Phosphorylation of PDX1 is required for its nuclear translocation and binding to target promoters (48). PDX1 phosphorylation occurs in response to PI3K/AKT signaling (49) and ERK1/2 (50). Blocking the PI3K/AKT pathway in pancreatic β cells reduces insulin content and insulin secretion (51). Overexpressing *Akt1* in pancreatic β cells increases β cell mass, proliferation, and cell size, which leads to improved glucose tolerance and insulin secretion (52, 53). A recent study reported that the improvements in glucose tolerance, β cell proliferation, and β cell mass induced by enhanced AKT signaling were blunted in PDX1-deficient mice (49). Furthermore, FOXO1 is an established upstream regulator of PDX1. FOXO1 acts as a repressor of FOXA2, which is known to activate the *Pdx1* promoter. Haploinsufficiency of FOXO1 reverses β cell failure in *Irs2^–/–^* mice through partial restoration of β cell proliferation and increased expression of *Pdx1* (54). In pancreatic cancer cells, inhibition of PI3K/AKT and MAPK/ERK pathways activates FOXO transcription factors, leading to cell cycle arrest and apoptosis (55). Moreover, in pancreatic β cells, MAPKs ERK1/2 have been shown to be the major expressed forms of ERKs, playing an essential role in mediating cell proliferation (56, 57). TRPM7 is a known regulator of the PI3K/AKT, SMAD, and ERK1/2 signaling pathways (29, 58). Our data suggest that TRPM7 kinase might directly or indirectly phosphorylate AKT and ERK1/2. Activation of the AKT and ERK1/2 pathways enhances PDX1 transcriptional activity, leading to compensatory β cell hypertrophy and proliferation. However, it is worth mentioning that AKT also induces proliferation of β cells through direct regulation of cyclin D1, cyclin D2, and CDK4 levels (59). Our results do not exclude the possibility that TRPM7 kinase might be involved in β cell cycle regulation and proliferation in a PDX1-independent manner. To further corroborate the concept that TRPM7 kinase regulates AKT/ERK signaling, we transfected MIN6 cells with *Trpm7* WT and *Trpm7^R/R^*. We found that overexpression of *Trpm7* WT in MIN6 cells enhances phosphorylation of ERK1/2 and AKT and leads to increases in insulin secretion. These results further support the notion that the detrimental glucoregulatory effects in *Trpm7^R/R^* mice are due to mitigated AKT/ERK signaling.

Obesity is a leading pathogenic factor for developing insulin resistance. Insulin resistance in obese individuals triggers a compensatory response in pancreatic islets. In this study, we provide evidence that TRPM7 kinase regulates insulin production and elicits an appropriate compensatory islet response to an obesogenic diet. Furthermore, the results from this study point to a potential link between TRPM7 kinase activity and the expression of critical genes required for insulin biosynthesis and cell cycle regulation. Therefore, we identify TRPM7 kinase as a critical cellular gatekeeper to preserve and improve β cell function under metabolically challenging circumstances.

## Methods

### Mouse strains and genotyping procedures.

MIP-Cre/ERT and *Trpm7^tm1Clph^* (*Trpm7^fl/fl^*) mice were obtained from The Jackson Laboratory. *Trpm7tm1.1Mkma* *C56BL/6* (K1646R, *Trpm7^R/R^*) mice were provided by Masayuki Matsushita (Okayama University Medical School, Okayama, Japan). *Trpm7^fl/fl^* mice (60) and *Trpm7^R/R^* mice (61) were reported previously. Mice were backcrossed to C57BL/6 (≥6 generations). Mice were housed in ventilated cages at the animal facility of the Walther Straub Institute of Pharmacology and Toxicology, LMU Munich, Munich, Germany. *Trpm7^fl/fl^* and MIP-Cre/ERT mice were bred to obtain age- and sex-matched homozygous *Trpm7^fl/fl^* MIP-Cre/ERT mice. To induce Cre activity in β cells of adult mice, 8-week-old male *Trpm7^fl/fl^* MIP-Cre/ERT mice were injected i.p. with tamoxifen in corn oil (2 mg/d/mouse for 5 consecutive days). Negative controls were *Trpm7^fl/fl^* MIP-Cre/ERT mice, which received just injections of corn oil. Heterozygous K1646R animals were bred to obtain age- and sex-matched homozygous WT and homozygous *Trpm7^R/R^* mice. For genotyping, DNA was extracted from ear fragments using the Mouse Direct PCR Kit (Biotool). DNA samples were analyzed by PCR using a set of allele-specific oligonucleotides (Metabion). Sequence information is provided in Supplemental Table 2. Genotyping of *Trpm7^fl/fl^* and *Trpm7^R/R^* mice was performed as previously described (3). Inheritance of MIP-Cre/ERT transgene was determined by PCR using the following conditions: 94°C for 2 minutes followed by 94°C for 15 seconds, 60°C for 15 seconds, 72°C for 10 seconds, last 3 steps repeated for 30 cycles, and 72°C for 2 minutes.

Male and female mice were fed chow diet or diabetogenic diet (Research Diets, D12451), containing 45% kcal from fat, beginning at 8 weeks of age. Mice were single- or group-housed on a 12-hour light/12-hour dark cycle at 22°C with free access to food and water. Mice were maintained under these conditions for a maximum of 36 weeks.

### Characterization of glucose homeostasis.

For the determination of glucose tolerance, 8- or 24-week-old mice (male and female) were fasted overnight (16 hours). Basal blood glucose was sampled, and glucose was administered as an intraperitoneal (i.p.) injection at a dose of 2 g/kg body weight (20% w/v d-glucose from MilliporeSigma in 0.9% w/v saline). Blood samples were obtained from the tail vein. Blood glucose levels were measured by glucometer (TheraSense FreeStyle) before (0 minutes) and at 15, 30, 60, and 120 minutes after injection. For the determination of insulin tolerance, mice were fasted for 4 hours at the onset of the light cycle and injected intraperitoneally with 0.75 units of insulin/kg body weight. Blood glucose levels were measured by glucometer (TheraSense FreeStyle) before (0 minutes) and at 15, 30, 60, and 120 minutes after injection. For investigation of blood parameters, blood was collected after euthanasia using EDTA-coated microvette tubes (Sarstedt), immediately cooled on ice, centrifuged at 2,000*g* and 4°C for 10 minutes, and plasma stored at −80°C. Plasma insulin was quantified by an insulin ELISA kit (ALPCO).

### Islet isolation and determination of insulin secretion.

Islets were isolated from 8- to 36-week-old male and female mice. Isolation of pancreatic islets was performed as previously described (62). In brief, pancreas was perfused by injection of 3 mM Collagenase-P (Roche) (0.3 mg/mL) in HBSS containing 25 mM HEPES and 0.5% (*w/v*) BSA into the common bile duct. Isolated islets were recovered for 48 hours in RPMI 1640 (Thermo Fisher Scientific) in humidified 5% CO_2_, at 37°C. After this period, islets were used for functional assessments. Before determination of insulin secretion, islets were equilibrated for 1 hour in KRB buffer (115 mM NaCl, 4.5 mM KCl, 1.2 mM KH_2_PO_4_, 2.6 mM CaCl_2_, 1 mM MgCl_2_, 10 mM HEPES, 20 mM NaHCO_3_, 0.2% *w/v* BSA, pH 7.4) with 2.8 mM glucose. Determination of insulin secretion from the islets was performed in 12-well plates containing 60 μL KRB (8 islets/well, 5 independent experiments performed in triplicate). After 1 hour preincubation in KRB with 2.8 mM glucose, islets were incubated for 1 hour in 20 mM glucose, 25 mM KCl, or 300 μM tolbutamide. Released insulin was measured in the supernatant using an insulin ELISA kit. Insulin content was determined from groups of 10 islets lysed in the protein extraction reagent M-PER (Thermo Fisher Scientific), using insulin ELISA kit.

### Calcium imaging.

Islets were loaded with 4 μM fluo-4 AM (Invitrogen) for 2 hours at room temperature in extracellular buffer containing 138 mM NaCl, 5.6 mM KCl, 2.6 mM CaCl_2_, 1 mM MgCl_2_, and 5 mM HEPES, pH 7.4. Changes in [Ca^2+^]_i_ were recorded by laser-scanning confocal microscopy using an LSM 510 Meta system (Zeiss) using a water immersion objective (63×/NA1.2). Individual cells were selected as “regions of interest” with the LSM software, and their calcium responses to the different stimuli were measured as alterations in fluo-4 emission intensity at 500–550 nm upon excitation with the 488 nm line of an argon laser. Eight-bit 512 × 512 pixel images were acquired every 5 seconds. Calculation of calcium oscillation frequency and amplitude is described in detail in Supplemental Methods.

### Electrophysiological recordings.

Whole-cell membrane currents were recorded using an EPC-9 amplifier (HEKA Electronics). Patch pipettes were pulled from glass capillaries GB150T-8P (Science Products) at a vertical Puller (PC-10, Narishige) and had resistances of 3 to 4 MΩ when filled with internal solution. The internal solution (0 Mg) consisted of (in mM) 120 Cs-glutamate, 8 NaCl, 10 HEPES, and 10 Cs-EDTA to chelate internal divalents (pH adjusted to 7.2 with CsOH). The extracellular solution contained (in mM) 140 NaCl, 2 MgCl_2_, 1 CaCl_2_, 10 HEPES, and 10 glucose (pH adjusted to 7.2 with NaOH). DVF (CaCl_2_ and MgCl_2_ were omitted from the external solution and 5 mM Na-EDTA was added) was directly applied onto the patch-clamped cell via an air pressure–driven (MPCU, Lorenz Meßgerätebau) application pipette. All solutions revealed an osmolality of 290 to 310 mOsm. Every 2 seconds voltage ramps of 50 ms duration spanning from –100 mV to +100 mV were applied from a holding potential of 0 mV using the PatchMaster software (HEKA Electronics). All voltages were corrected for a liquid junction potential of 10 mV, and currents were filtered at 2.9 kHz and digitized at 100 μs intervals. Before each voltage ramp, capacitive currents and series resistance were determined and corrected by the EPC9 automatic capacitance compensation. Inward and outward currents at –80 and +80 mV were extracted from each individual ramp current recording, and amplitudes were plotted versus time. IVs were extracted at indicated time points. To obtain the net developing current (I_net_), basic currents (I_min_) were subtracted from single IVs. All currents were normalized to the initial size, i.e., capacitance of the cell to obtain current densities (pA/pF).

### Morphological analysis.

Standard hematoxylin and eosin staining on 10 μm cryosections of islets and immunofluorescence staining of whole islets were performed to assess pancreatic islet morphology. Antibodies and their working dilutions are listed in Supplemental Table 3. Digital imaging fluorescence microscopy of the pancreas was performed using a scanning platform (MetaSystems) with an Imager Z.2 microscope (Carl Zeiss MicroImaging, Inc.). Quantitative image analysis of islet morphology was performed using ImageJ (NIH). Size of β cells size was measured by imaging randomly selected cells at 400× and determined as mean individual β cell cross-sectional area for at least 5 islets per animal using ImageJ software. For the mean individual β cell cross-sectional area, the insulin-positive area of each islet was divided by the number of nuclei within the insulin-positive area. Investigators followed a blinded protocol during analysis.

### Western blot.

Western blot analysis was performed as previously described (63). A total of 20 μg of protein was loaded, resolved on 8%–12% Tris-HCl SDS-PAGE, and blotted onto a nitrocellulose membrane (Amersham Biosciences). Membranes were blocked for 1 hour using 5% BSA or nonfat dried milk diluted in Tris-buffered saline with 0.1% Tween 20 detergent at room temperature and incubated with primary antibodies (Supplemental Table 3) at 4°C for 16 hours. After washing, membranes were incubated with HRP-conjugated secondary antibodies (Supplemental Table 3) for 1 hour at room temperature. Immunobound antibody was visualized with an enhanced chemiluminescence kit (GE Healthcare Europe). ChemiDoc MP Imaging System (Bio-Rad) was used for chemiluminescence detection. For the loading control, membranes were stripped and incubated with an antibody against ERK2 or histone H3 for approximately 16 hours at 4°C.

### RNA isolation.

RNA was extracted from pancreatic islets using the RNeasy Mini Kit (QIAGEN), following the manufacturer’s instructions. cDNA was prepared using QuantiTect Reverse Transcription Kit (QIAGEN), according to the manufacturer’s protocol.

### qRT-PCR.

Real-time PCR was performed in triplicate with a Bio-Rad iCycler by cycling 40 times using the following conditions: 95°C for 10 seconds, 60°C for 45 seconds. Primers were designed using Primer3 as above and tested for linear amplification using serial dilutions of cDNA before use on experimental samples. Sequence information is provided in Supplemental Table 4.

### Measurement of β cell proliferation and apoptosis.

Pancreatic slices were prepared from *Trpm7^R/R^* and control littermates after 16 weeks of chow or HFD. To study β cell proliferation, pancreatic islets were costained for insulin and Ki67. Ki67-insulin double-positive cells were counted and divided by the total number of insulin-positive cells per pancreatic section. To investigate β cell apoptosis, ApopTag Red In Situ Apoptosis Detection Kit was used according to the manufacturer’s (Merck) instructions. TUNEL-insulin double-positive cells were counted and divided by the total number of insulin-positive cells per pancreatic section.

### RNA-Seq studies.

RNA-Seq data have been uploaded to the NCBI’s Gene Expression Omnibus under the accession number GSE218030 (https://www.ncbi.nlm.nih.gov/geo). Total RNA was extracted from isolated pancreatic islets of *Trpm7^R/R^* and their control littermates, which had been maintained on an HFD for 16 weeks. Template amplification and clustering were performed on the NovaSeq 6000 (Illumina) applying the exclusion amplification (ExAmp) chemistry. The ExAmp workflow is a proprietary Illumina method and ensures that only single DNA templates are bound within single wells of the patterned NovaSeq flow cells and are almost instantaneously amplified. Cluster generation and sequencing were operated under the control of the NovaSeq Control Software v1.6.0. The *P* value of a pairwise comparison was derived from the Wald test. To control the false-positive rate, FDR-corrected (64) as well as Bonferroni-corrected *P* values were calculated, where FDR is the proportion of false-positive hits among all positive hits. A gene or transcript is classified as upregulated or downregulated in a specific comparison if its FDR-corrected *P* value is ≤0.05 and its fold change is ≥2.

### Bio-Plex Pro cell signaling assay.

Murine pancreatic islets were washed and lysed in MILLIPLEX MAP Lysis Buffer (Merck). Protein content was measured using Pierce BCA Protein Assay Kit (Thermo Fisher Scientific, catalog 23225). Samples were stored at −80°C. Collected samples were processed and assayed according to manufacturer’s instructions specific for MILLIPLEX MAP TGFβ Signaling Pathway Magnetic Bead 6-Plex kit (Merck, catalog 48-614MAG), and MILLIPLEX MAP β-Tubulin Total Magnetic Bead MAPmate (Merck, catalog 46-713MAG).

### Cell culture.

Mouse WT and kinase-dead TRPM7 in pIRES-EGFP vector were reported previously (65). MIN6 cells were provided by Per-Olof Berggren and Barbara Leibiger, Karolinska Institutet, Stockholm, Sweden. MIN6 cells were grown at 37°C and 5% CO_2_ in DMEM (MilliporeSigma) supplemented with 10% FBS (Thermo Fisher Scientific), 100 U/mL penicillin and 100 μg/mL streptomycin (MilliporeSigma), and 75 μM β-Mercaptoethanol (Gibco). Cells with approximately 60% confluence in 96-well plates or 6 cm dishes were transiently transfected by 0.1 or 2 μg cDNAs, respectively. TurboFect was used as a transfection reagent (Thermo Fisher Scientific). GIIS was measured 48 hours after transfection in 96-well plates. Cells were harvested 48 hours after transfection from 6 cm dishes for Western blotting.

### Statistics.

Data were expressed as mean ± SEM. *P* value less than 0.05 was considered significant. Graph presentations, curve fittings, statistics, and *P* values were obtained by Prism software (version 9.0.1; GraphPad). For comparison of 2 groups, *P* values were calculated by the unpaired 2-tailed Student’s *t* test for parametric or Mann-Whitney test for nonparametric distribution. For 3 or more groups, 1-way ANOVA with Bonferroni’s multiple comparison were used for parametrically distributed data. Glucose and insulin tolerance tests were compared using 2-way ANOVA with Bonferroni’s multiple comparison.

### Study approval.

All animal experiments were performed in accordance with the EU Animal Welfare Act and were approved by the District Government of Upper Bavaria, Munich, Germany, on animal care (permit no. 55.2-2532.Vet_02-19-035).

## Author contributions

NK designed and conducted experiments, analyzed and interpreted data, prepared figures, and wrote the manuscript. A Beck, KR, PB, KJ, SFS, A Belkacemi, PCFS, HS, and TP conducted experiments, analyzed and interpreted data, and edited the manuscript. PSR, AN, A Breit, VC, TDM, and SZ interpreted data and edited the manuscript. TG directed the project, designed experiments, interpreted data, and edited the manuscript.

## Supplementary Material

Supplemental data

## Version 1 12/27/2022

In-Press Preview

## Version 2 02/08/2023

Electronic publication
