# Supplementary material for: TRPM7 kinase is required for insulin production and compensatory islet responses during obesity
Source: JCI Insight. 2023 Feb 8;8(3):e163397. doi: 10.1172/jci.insight.163397 (PMC9977431; doi:10.1172/jci.insight.163397)
Supplement: Supplemental data [file jciinsight-8-163397-s083.pdf]

TRPM7 kinase is required for insulin production and compensatory islet responses during obesity

Noushafarin Khajavi[1], Andreas Beck[2], Klea Riçku[1], Philipp Beyerle[1], Katharina Jacob[1], Sabrina F. Syamsul[1], Anouar Belkacemi[2], Peter S. Reinach[3], Pascale C F Schreier[1], Houssein Salah[2], Tanja Popp[4], Aaron Novikoff[5, 6], Andreas Breit[1], Vladimir Chubanov[1], Timo D. Müller[5, 6], Susanna Zierler[1, 7], Thomas Gudermann[1, 8]

[1]Walther Straub Institute of Pharmacology and Toxicology, LMU Munich, Germany; [2]Institut für Experimentelle und Klinische Pharmakologie und Toxikologie, Universität des Saarlandes, Homburg, Germany; [3]Wenzhou Medical University, Ophthalmology Department, Wenzhou, P.R. China; [4]Bundeswehr Institute of Radiobiology, Munich, Germany; [5]Institute of Diabetes and Obesity, Helmholtz Center Munich, Neuherberg, Germany; [6]German Center for Diabetes Research (DZD), Neuherberg, Germany; [7]Institute of Pharmacology, Medical Faculty, Johannes Kepler University Linz, Linz, Austria; [8]German Center for Lung Research, Munich, Germany

Corresponding authors:

Prof. Dr. Thomas Gudermann
Walther Straub Institute of Pharmacology and Toxicology Faculty of Medicine, LMU Munich
Goethestr. 33, 80336 Muenchen
Phone: +49 (0)89 2180 75700/2
E-mail: Thomas.Gudermann@lrz.uni-muenchen.de

Dr. Noushafarin Khajavi
Walther Straub Institute of Pharmacology and Toxicology Faculty of Medicine, LMU Munich
Goethestr. 33, 80336 Muenchen
Phone: +49 (0)89 2180 757748
E-mail: Noushafarin.Khajavi@lrz.uni-muenchen.de

Calculation of calcium oscillation frequency and amplitude

The algorithm is based on three stages that parse over an array of data (the "input data array" or IDA).

First stage is marking all areas of the IDA in a second array (the "rising slope indicator array" or RSIA). For each value in the IDA, if the current value in the IDA is higher or equal to the previous value, then the input data is rising and will be marked in the RSIA with a value higher than zero (10 in this case to be more visible in chart). If the current value in the IDA is lower than the previous value, then the data is falling, and it will be marked with a 0 in the RSIA.

Second stage is looking at the areas in the RSIA where a value higher than zero is found and writing the results in a third array (the "accumulator array" or AA). For each value in the RSIA, if the value is higher than zero, then the difference between the current value of the IDA and the previous value of the IDA, plus the previous value of the AA are written in the current AA position.

Third stage is used to indicate where the peaks are in the data in a fourth array (the "peak array" or PA). For each value in the RSIA, if the current value of the RSIA is smaller than the previous value of the RSIA then this was the peak and the current value of the RSIA will be written in the PA. If the current value of the RSIA is larger than the previous value of the RSIA then the peak has not been reached yet and the value zero will be written in the PA.

*Note: all three stages begin processing at the second value because it always needs to compare the current data with a previous one. All other arrays (RSIA, AA and PA) will be initialized with zero in their respective first values.

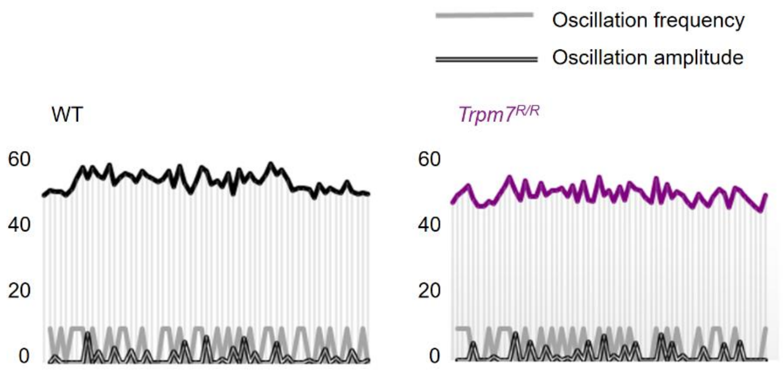

Suppl. Table 1. Relevant upregulated genes in *Trpm7*$^{R/R}$ mice

Function	Gene Name	Chromosome	Log$_2$ fold change	Fold change	P-value	FDR p-value
Insulin production and maturation	*Ins2*	7	-2.48234	-5.58804	3.51E-08	0.000112
	MafA	15	-2.39025	-5.2425	4.72E-07	0.000375
	Ins1	19	-2.19683	-4.58471	5.45E-08	0.000124
	Nkx6-1	5	-1.64286	-3.12284	0.001492	0.095847
	Pdx1	5	-1.52139	-2.87067	0.000223	0.025192
	Cpe	8	-1.51368	-2.85537	0.00011	0.015111
	Pcsk2	2	-1.4759	-2.78156	0.000372	0.036756
	Neurod1	2	-1.45185	-2.73558	0.000628	0.054728
	Pcsk2	2	-1.4759	-2.78156	0.000372	0.036756
Cell cycle regulation and proliferation	*Ccnd2*	6	-1.74845146	-3.35997724	1.14791E-05	0.002684
	Cdk5rap3	11	-1.68616399	-3.21799926	0.000143	0.018181
	Cdk7	13	-1.58838201	-3.0071191	0.001550	0.097273
	Cdk4	10	-1.53534518	-2.89857775	0.001847	0.109914
	Cdk19	10	-1.03435704	-2.04820063	0.019330	0.4692235
	Tubb2b	13	-2.34724013	-5.0884989	0.0474312	0.6904990
	Pimreg	11	-2.05969495	-4.16898143	0.0257293	0.5363203
	Cirp	10	-1.2112224	-2.31533734	0.024131	0.5214590
	Mll5	5	-0.97183433	-1.96133277	0.017508	0.443330
ER-related	*Atf4*	15	-2.03524426	-4.09892121	1.3936E-06	0.00079158
	Hspa5	2	-1.78298392	-3.44137218	2.4207E-06	0.00097726
	Pdia4	6	-1.43366452	-2.70131994	0.01258935	0.37390722
	Pdia6	12	-1.00312872	-2.00434203	0.01523545	0.416702

Suppl. Table 2. Relevant invariant genes in *Trpm7*$^{R/R}$ mice

Function	Name	Chromosome	Log$_2$ fold change	Fold change	P-value	FDR p-value
Glucose sensing and exocytosis machinery	*Syt5*	7	-0.46425	-1.3796	0.372308	0.959028
	Stxbp1	2	-0.18323	-1.13542	0.671802	0.991372
	Cacna1d	14	0.141117	1.102758	0.722141	0.99213
	Cacna1c	6	0.117214	1.084638	0.752066	0.99213
	Stx1a	5	-0.48575	-1.40031	0.632985	0.979122
	Snap25	2	0.049579	1.034963	0.895555	0.99213
	Syt9	7	0.179614	1.13258	0.642769	0.981937
	Cacnb1	11	-0.38348	-1.30449	0.620004	0.978128
	Glud1	14	0.064059	1.045403	0.870745	0.99213
	Slc2a2	3	-0.68437	-1.607	0.085683	0.837582
	Gck	11	-0.71194	-1.63801	0.244983	0.959028
	G6pc	11	0.051344	1.03623	0.890324	0.99213
	Mpc1	17	-0.35754	-1.28124	0.352265	0.959028

Suppl. Table 3. Antibody descriptions

**Antigen**	**Host Species**	**Dilution**	**Source**	**Catalog Number**
Glucagon	Mouse	1:1000	Sigma	G2654
Insulin	Guinea pig	Ready to use	Dako	IR002
Ki67	Rabbit	1:500	Abcam	Ab 15580
PDX1	Rabbit	1:500	Abcam	Ab134150
Histon H3	Rabbit	1:10000	Abcam	Ab1791
ERK2	Rabbit	1:10000	Santa Cruz	sc-154
P-ERK	Rabbit	1:1000	ThermoFisher	44-680G
Tot. ERK	Mouse	1:1000	ThermoFisher	13-6200
P-AKT (S473)	Rabbit	1:1000	Cell Signaling	9271
Tot. AKT	Rabbit	1:1000	Cell Signaling	9272
Anti-M7d (2C7)	Mouse	1:1000	doi.org/10.7554/eLife.68544	
Alexa Fluor 488 goat anti-guinea pig	Goat	1:1000	ThermoFisher	A11073
Alexa Fluor 594 goat anti-guinea pig	Goat	1:1000	ThermoFisher	A11076
Alexa Fluor 647 goat anti-rabbit	Goat	1:1000	ThermoFisher	A21245
Alexa Fluor 488 goat anti-mouse	Goat	1:1000	ThermoFisher	A11001
Goat anti-Guinea Pig IgG (H+L) Secondary Antibody, HRP	Goat	1:1000	ThermoFisher	A18769
Goat anti-Rabbit IgG (H+L) Secondary Antibody, HRP	Goat	1:1000	ThermoFisher	A31460

Suppl. Table 4. qRT-PCR primers

Gene	Primer sequence	Amplicon (bp)
Ins2	Forward: 5’ CTGGCCCTGCTCTTCCTCTGG<br>Reverse: 5’ CTGAAGGTCACCTGCTCCCGG	204
Pdx1	Forward: 5’ CCCCAGTTTACAAGCTCGCT<br>Reverse: 5’ CTCGGTTCCATTCGGGAAAGG	177
Mafa	Forward: 5’ AGGAGGAGGTCATCCGACTG<br>Reverse: 5’ CTTCTCGCTCTCCAGAATGTG	113
Gapdh	Forward: 5’ GTGGAGTCATACTGGAACATGTAG<br>Reverse: 5’ AATGGTGAAGGTCGGTGTG	150
Trpm7 kinase	Forward: 5’ AATGGGAGGTGGTTT ACG<br>Reverse: 5’ CTCAGATCACAGCTTACAGTC A	205
Trpm7	Forward: 5’ AGTAATTCAACCTGCCTCAA<br>Reverse: 5’ ATGGGTATCTCTTCTGTTATGTT	287
Ins1<br>*Cre*	Forward: 5’ TGGACTATAAAGCTGGTGGGCAT<br>Reverse: 5’ TGCGAACCTCATCACTCGT	230

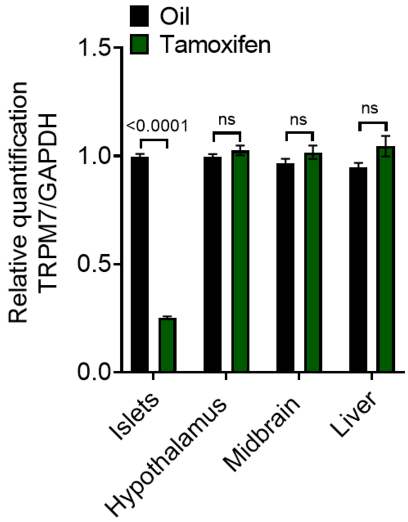

Supplemental Figure 1 *Trpm7* gene expression analyzed by qRT-PCR from RNAs isolated from pancreatic islets, hypothalamus, midbrain and liver of *Trpm7*$^{flox/flox}$;MIP-Cre/ERT mice 28-wks post recombination (n = 4 mice per genotype). Negative controls were corn oil-injected *Trpm7*$^{flox/flox}$;MIP1-CreERT mice. GAPDH was used as a reference transcript. Data are given as means ± S.E.M. and statistical differences were assessed by unpaired two-tailed Student's t-test. *P* values are shown above the bars. (ns, not significant)

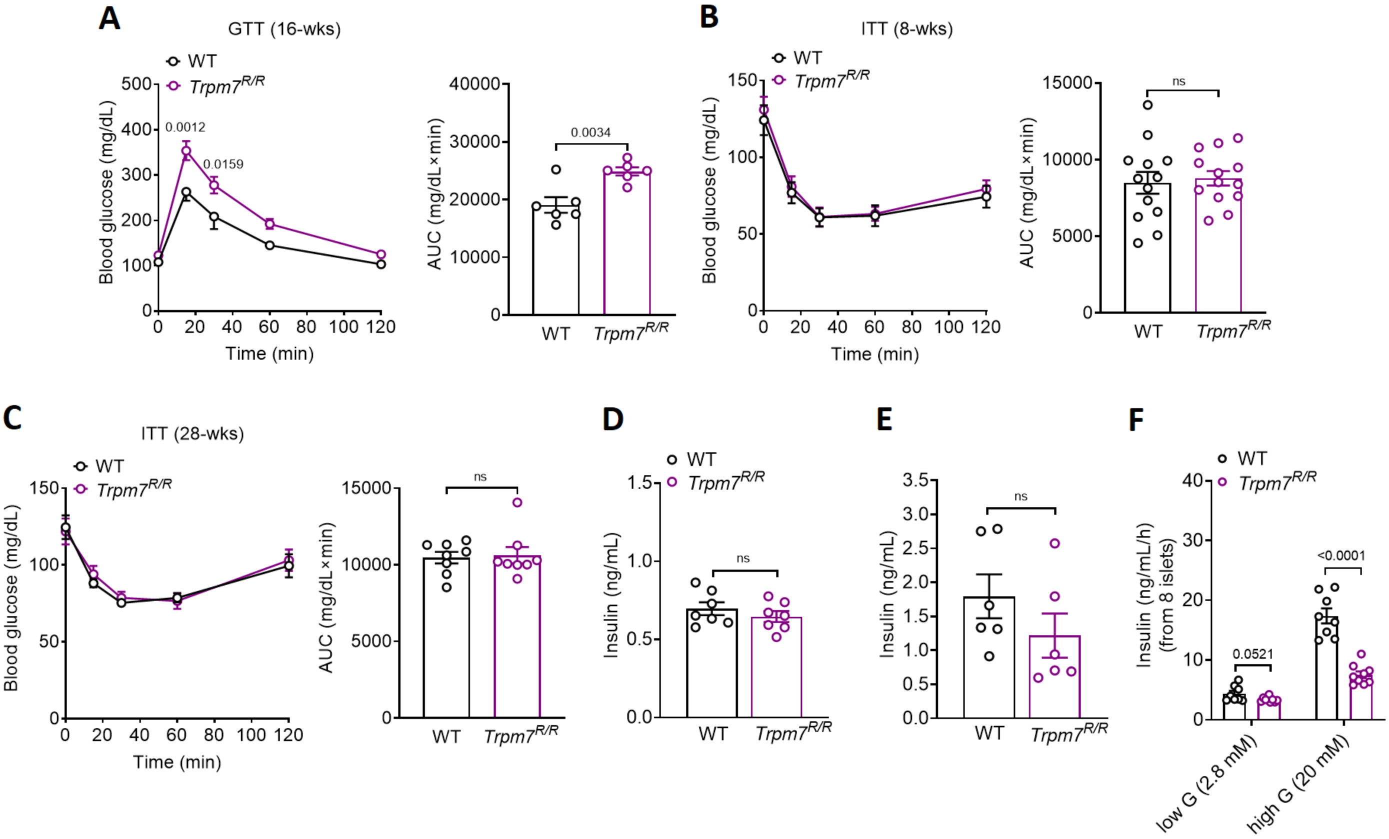

Supplemental Figure 2 (**A**) Adult mice (*Trpm7*$^{R/R}$ and control littermates) maintained on a chow diet. For glucose tolerance test (GTT), mice were fasted overnight (*n* = 6 mice per genotype). Blood glucose levels (mg/dL) before and within 2 hours after i.p. injection of glucose (2 g/kg of body weight) in WT and *Trpm7*$^{R/R}$ mice (left panels) and area under the curves (AUC in mg/dL × min; right panels) after 16-wks. (**B**, **C**) For insulin tolerance test (ITT) mice were fasted for 4 hours at the onset of the light cycle ($n \geq 8$ mice per genotype). Blood glucose levels (mg/dL) before and within 2 hours after i.p. injection of insulin (0.75 U/kg of body weight) in WT and *Trpm7*$^{R/R}$ mice (left panels) and AUC (mg/dL × min; right panels) after 8- 9- wks (B) and 28-wks (C). (**D**, **E**) Plasma insulin levels (ng/mL) in freely fed ($n \geq 6$ mice per genotype) in male and female *Trpm7*$^{R/R}$ and control littermate mice were measured after 8- 9- wks (D) and 28-wks (E). (**F**) Insulin secretion (ng/mL/h/8 islets) in isolated islets of male and female *Trpm7*$^{R/R}$ and control littermate mice at 28-wks of age. Islets were incubated for 1 hour in the presence of low glucose (2.8 mM) or high glucose (20 mM) ($n \geq 4$ mice per genotype, measured in duplicate). Data show means ± S.E.M., and statistical differences were assessed by two-way ANOVA (A, B, C left) or unpaired two-tailed Student's t-test (A, B, C right, D, E, F). Circles in bar graphs represent single values. *P* values are shown above the bars. (ns, not significant)

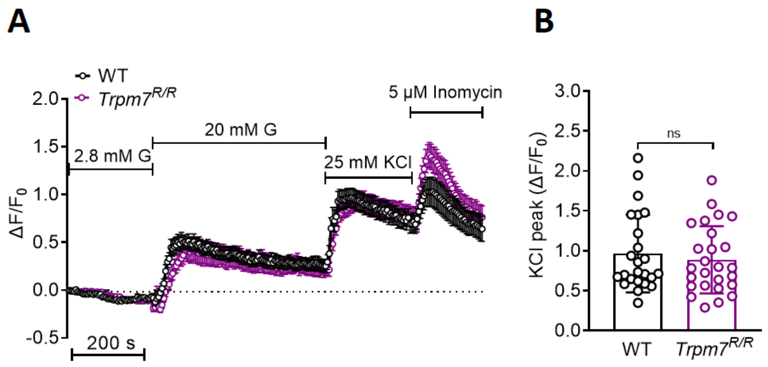

Supplemental Figure 3 (**A**) Alterations in [Ca^{2+}]$_i$ were monitored in pancreatic islets loaded with fluo-4 AM after increasing the glucose from 2.8 to 20 mM and application of 25 mM KCl in WT (black; *n* = 24 cells from 3 mice) and *Trpm7$^{R/R}$* (purple; *n* = 26 cells from 3 mice) islets. Ionomycin (5 µM) was used as a positive control. (**B**) Average of Ca^{2+} influx peaks assessed from baseline after stimulation with KCl in WT and *Trpm7$^{R/R}$* β-cells. The cells which displayed no increase in [Ca^{2+}]$_i$ in response to high potassium concentration are excluded from the results. Data shown are means ± S.E.M. (circles in bar graphs represent single values) and statistical differences were assessed by unpaired two-tailed Student's t-test. (ns, not significant)

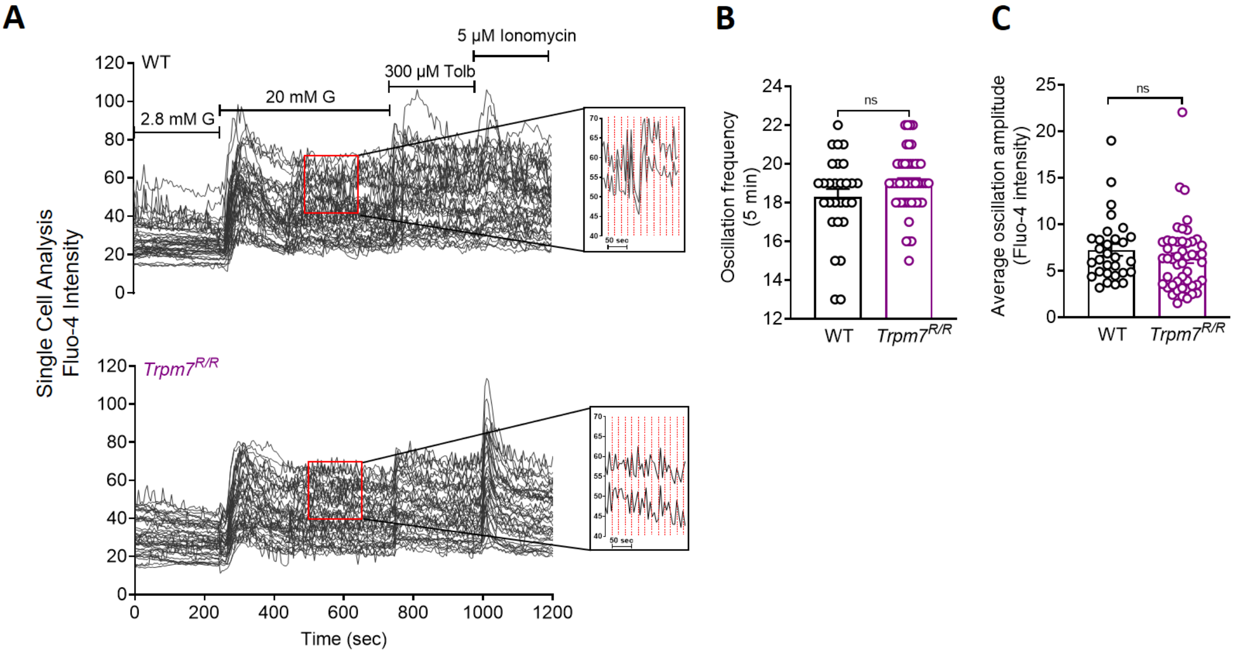

Supplemental Figure 4 (**A**) Individual traces of fluo-4 intensity of single islet cells from WT and *Trpm7*$^{R/R}$ mice, elicited by excitation at 480 nm (indicative of $[Ca^{2+}]_i$). Inserts show amplified sections indicating the regions from which data in the right panels was taken. (**B**) Representative changes in frequency of $[Ca^{2+}]_i$ oscillations in 5 min following an initial peak after 20 mM glucose stimulation ($n \geq 27$ cells from 3 mice per genotype). (**C**) Average $[Ca^{2+}]_i$ oscillation amplitude calculated based on alteration in fluo-4 intensity in single cells following an initial peak after 20 mM glucose stimulation ($n \geq 27$ cells, 3 mice per genotype). Data shown are means ± S.E.M. (circles in bar graphs represent single values) and statistical differences were assessed by unpaired two-tailed Student's t-test. (ns, not significant)

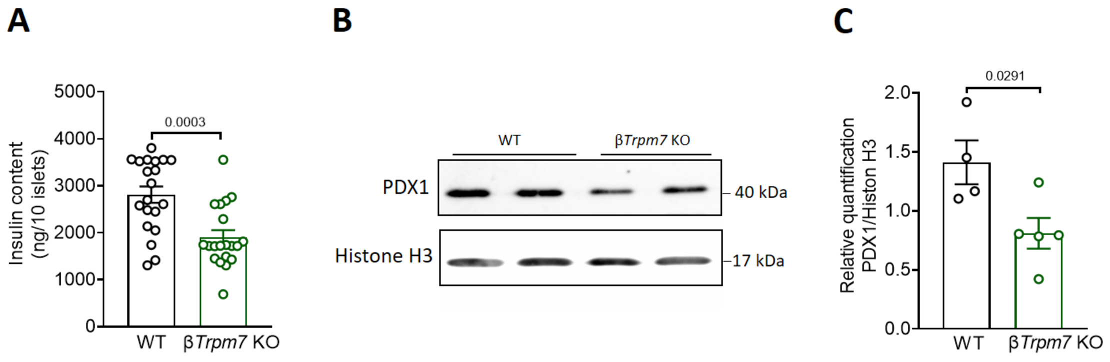

Supplemental Figure 5 (**A**) Total insulin content of pooled isolated islets from WT versus β*Trpm7* KO mice after 28-wks of tamoxifen-induced recombination. At least 20 groups of 10 size-matched WT and *Trpm7*$^{R/R}$ islets were compared. (**B** and **C**) Western blot detection of the PDX1 in lysates of purified islets from WT and β*Trpm7* KO mice after 28-wks of tamoxifen-induced recombination ($n \geq 4$, at least 3 mice per genotype). Histone H3 was used as loading control. Data are given as means ± S.E.M. (circles in bar graphs represent single values) and statistical differences were assessed by Mann-Whitney test (A) or unpaired two-tailed Student's t-test (C). *P* values are shown above the bars.

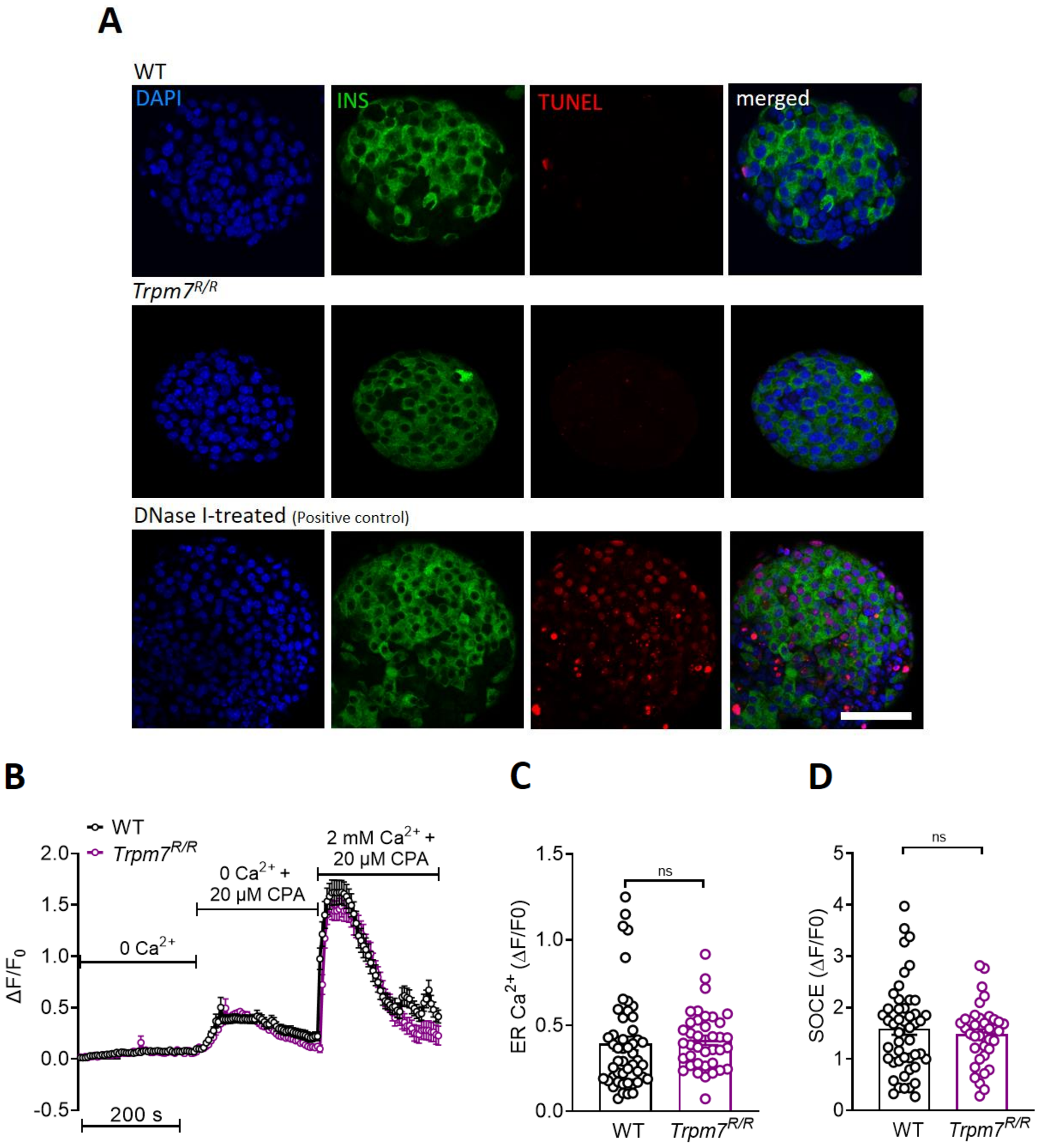

Supplemental Figure 6 (**A**) TRPM7 kinase disruption does not induce apoptosis in pancreatic β-cells. TUNEL staining of isolated islets from WT and *Trpm7$^{R/R}$* mice. DNase I-treated islets are used as positive control. TUNEL staining is shown in red, insulin staining is shown in green, and nuclei (DAPI) are shown in blue, (*n* = 50 slides, 3 mice per genotype). The scale bar represents 100 µm. (**B**) $[Ca^{2+}]_i$ responses following store depletion in the presence of 20 µM CPA in the absence of extracellular Ca^{2+} and re-addition of 2 mM Ca^{2+} (SOCE) in WT (n = 49 cells from 3 mice per genotype) and *Trpm7$^{R/R}$* islets (*n* = 38 cells from 3 mice per genotype). (**C**) ER Ca^{2+} levels were indirectly estimated by quantitating the $\Delta F/F_0$ in response to CPA. (**D**) Average of Ca^{2+} influx peaks assessed from baseline and obtained from SOCE measurements in β-cells. Graphs in (C) and (D) were generated from data shown in (B). Data shown are means ± S.E.M. (circles in bar graphs represent single values) and statistical differences were assessed by unpaired two-tailed Student's t-test. (ns, not significant)

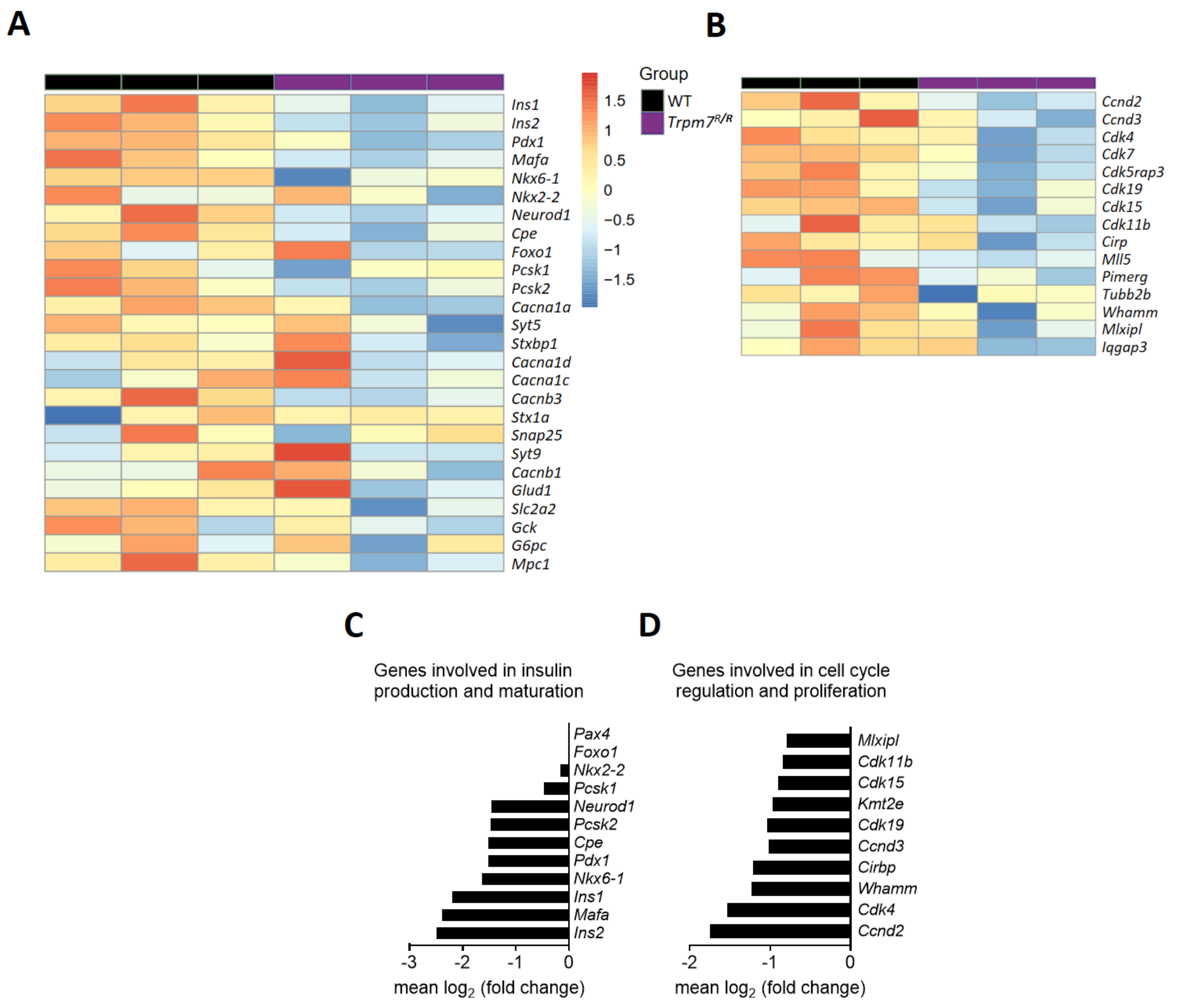

Supplemental Figure 7 (**A**, **B**) Heat maps displaying color-coded expression levels of differentially expressed genes. Islet RNA was collected from *Trpm7*$^{R/R}$ mice and the control littermates that had been maintained on a HFD for ~16-wks (*n* = 3 mice per genotype). The heat maps show the differential expression of genes involved in (A) insulin production, glucose sensing and exocytosis machinery and in (B) cell cycle and cyclin-dependent protein kinase activity. Summary of the genes involved in (**C**) insulin production and (**D**) cell cycle regulation that were downregulated in *Trpm7*$^{R/R}$. DEGs are expressed as $\log_2$ fold change over control with an adjusted *P* value for each gene.

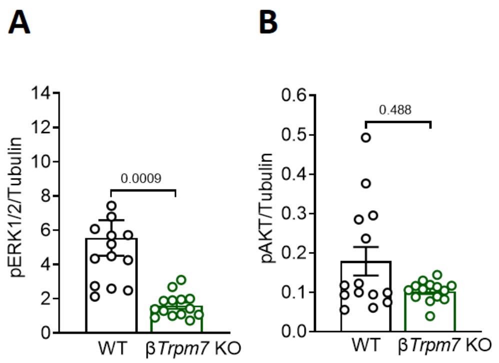

Supplemental Figure 8 (**A, B**) Assessment of the activity of the cell signaling molecules ERK1/2, and AKT using multi-Plex assay and phospho-specific antibodies on lysates of isolated islets from WT and β*Trpm7* KO mice (*n* = 7, measured in duplicates, 7 mice per genotype) under 16-wks of HFD. Data are normalized to Tubulin content. Data show means ± S.E.M. and statistical differences were assessed by unpaired two-tailed Student's t-test (A, B). Circles in bar graphs represent single values. *P* values are shown above the bars.

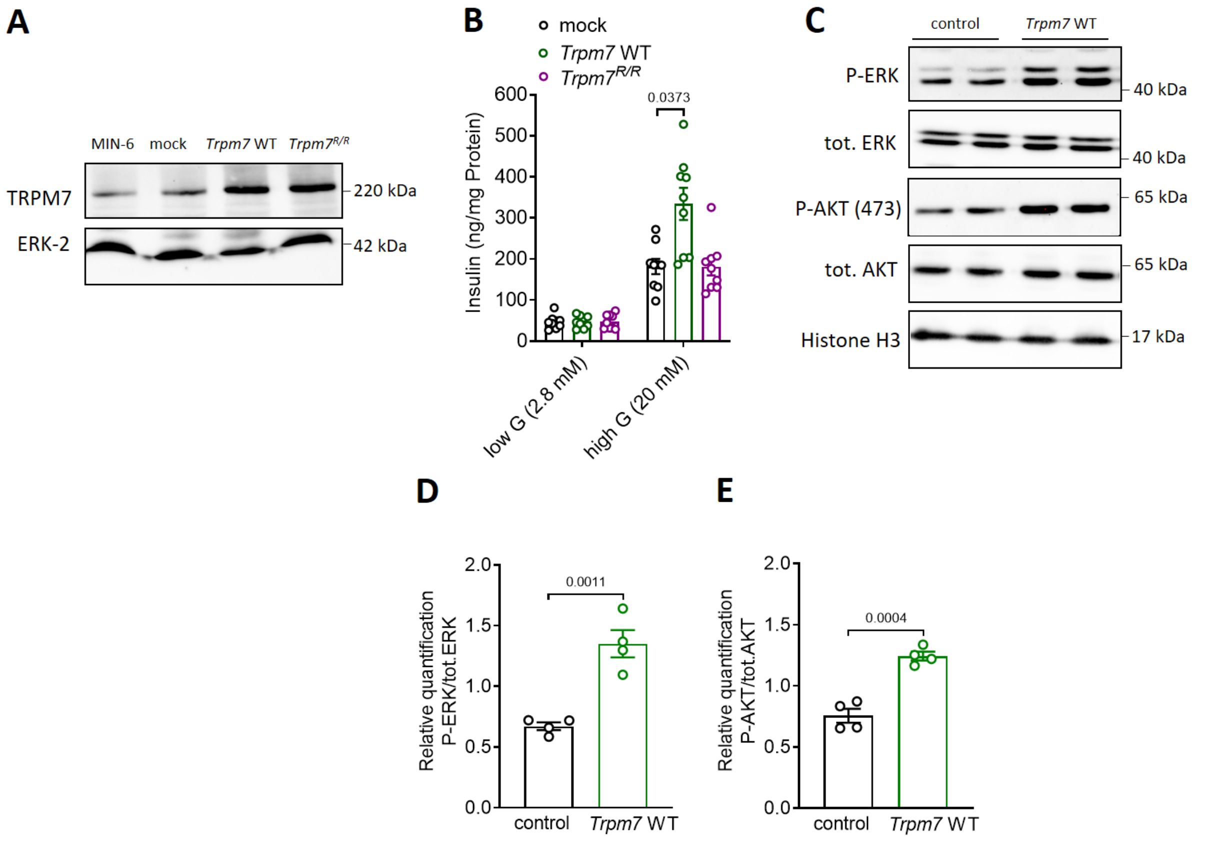

Supplemental Figure 9 (**A**) Western blot detection of the TRPM7 in MIN6 cells transiently transfected with *Trpm7* WT, *Trpm7*$^{R/R}$ or with empty vector. ERK2 was used as loading control. (**B**) Insulin secretion (ng/mg protein) was measured in MIN6 cells transfected with *Trpm7* WT, *Trpm7*$^{R/R}$ in pIRES-EGFP vector or empty vector (mock) 48 hours after transfection. MIN6 cells were incubated for 1 hour in the presence of low glucose (2.8 mM), high glucose (20 mM). GIIS was measured at least 3 times in duplicate (**C**) Lysates from MIN6 cells that had been treated with *Trpm7* WT, *Trpm7*$^{R/R}$ or with empty vector were subjected to Western blotting studies. Blots were probed with the indicated antibodies. (**D**, **E**) Quantification of the Western blotting data shown in (C). Each experiment was performed at least 3 times. Data are given as mean ± S.E.M. (circles in bar graphs represent single values) and statistical differences were assessed by unpaired two-tailed Student's t-test. *P* values are shown above the bars.
